# Design, Synthesis, and Biological Evaluation of Novel Benzofuran Derivatives Bearing *N*-Aryl Piperazine Moiety

**DOI:** 10.3390/molecules21121684

**Published:** 2016-12-09

**Authors:** Yulu Ma, Xi Zheng, Hui Gao, Chunping Wan, Gaoxiong Rao, Zewei Mao

**Affiliations:** 1 School of Traditional Chinese Medicine, Yunnan University of Traditional Chinese Medicine, Kunming 650500, China; Rao_gx@163.com; 2 Central Laboratory, The No. 1 Affiliated Hospital of Yunnan University of Traditional Chinese Medicine, Kunming 650021, China; zhengxi138@163.com; 3 Key Laboratory of Medicinal Chemistry for Natural Resource Education Ministry, School of Chemical Science and Technology, Yunnan University, Kunming 650091, China; 22014000171@mail.ynu.edu.cn

**Keywords:** benzofuran, *N*-aryl piperazine moiety, anti-inflammatory activity, anticancer activity

## Abstract

A series of novel hybrid compounds between benzofuran and *N*-aryl piperazine have been synthesized and screened in vitro for anti-inflammatory activity in lipopolysaccharide (LPS)-stimulated RAW-264.7 macrophages and for anticancer activity against three human tumor cell lines. The results demonstrated that derivative **16** not only had inhibitory effect on the generation of NO (IC_50_ = 5.28 μM), but also showed satisfactory and selective cytotoxic activity against human lung cancer line (A549) and gastric cancer cell (SGC7901) (IC_50_ = 0.12 μM and 2.75 μM, respectively), which was identified as the most potent anti-inflammatory and anti-tumor agent in this study.

## 1. Introduction

**Scheme 1:**
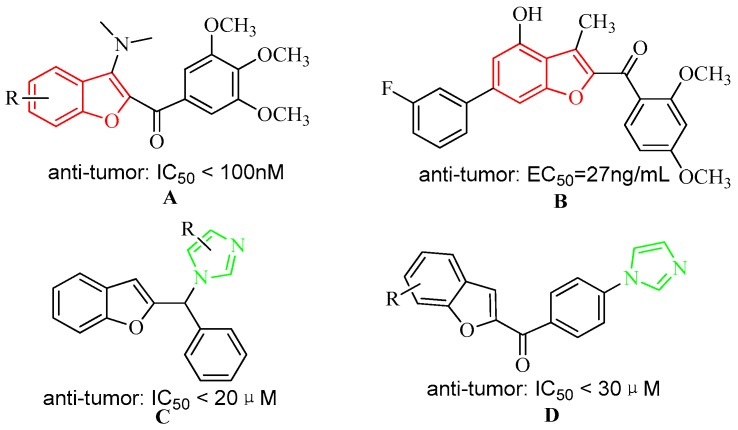
Structures of biological benzofuran agents.

Natural and synthetic benzofuran derivatives are a class of important organic compounds with a broad range of biological activities, such as antioxidant, anti-inflammatory, antibacterial, antitumor, and so on [1,2,3,4,5,6,7,8,9,10,11]. Recently, benzofuran derivatives have attracted considerable interest for their versatile properties in chemistry and pharmacology. In addition, 2-benzoylbenzofuran compounds have been identified to show potent cytotoxic activities, as exemplified in Scheme 1. 2-benzoylphenyl benzofuran compound (**A**) [12] and 2-(2,4-dimethoxybenzoyl)-phenyl benzofuran derivative (**B**) [13] showed potent anticancer activities. Furthermore, synthetic 2-benzylbenzofurane-imidazole hybrid (**C**) [14] and 2-(4-imidazoyl benzoyl)benzofuran (**D**) [15] were attractive with excellent anti-inflammatory and cytotoxic activities (Scheme 1).

Nitrogen heterocycles are an important class of compounds having versatile biological activities, which are used in drug design and synthesis, generally as active units [16,17]. Piperazine—one of the most biological active moieties—represents a series of important organic compounds that make up the core structures in medicines and have been widely used in the development of drug molecular design [18,19].

In previous work, we reported that benzofuran compounds containing *N*-heterocyclic moieties displayed potent cytotoxic activities [20], and the hybrids between chalcone and *N*-aryl piperazine bearing acetophenone showed potent anti-inflammatory and anticancer activities [21,22]. In addition, the acetophenone substituent was vital for modulating cytotoxic activities.

**Scheme 2:**
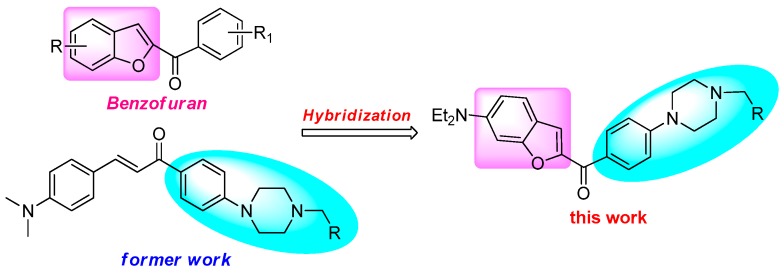
Designed strategy of benzofuran hybrids.

Based on these results, in the present research, we have designed and synthesized novel hybrids towards the recombination of benzofuran and *N*-aryl piperazine (Scheme 2). In order to study the structure–activity relationship (SAR) of hybrid compounds, various R-X were selected, including α-bromoacetophenone, benzyl bromide, and alkyl bromide. The potent in vitro anti-inflammatory activity in lipopolysaccharide (LPS)-stimulated RAW-264.7 macrophages and anticancer activities of compounds against a panel of human cancer cell lines (A549, Hela, and SGC7901) were evaluated, respectively.

## 2. Results

### 2.1. Chemistry

**Scheme 3:**
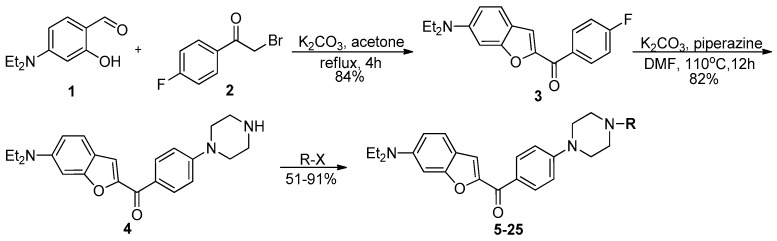
Synthetic routes of hybrid compounds.

The general synthetic route used to synthesize hybrid compounds is outlined in Scheme 3. Treatment of commercial 5-diethylaminosalicylaldehyde (**1**) with 2-bromo-4′-fluoro acetophenone (**2**) gave the 2-phenzoylbenzofuran compound (**3**) in the presence of K_2_CO_3_ in refluxing acetone. Then, the key benzofuran–piperazine intermediate (**4**) was prepared by substitution with piperazine from compound (**3**) in the presence of K_2_CO_3_ at 110 °C in DMF. With the desired intermediate (**4**) in hand, we began the synthesis of amines by treatment of compound (**4**) with several commercially available R-X. To get further insight into the structure–activity relationship, the tertiary amines (**5**–**25**) were prepared with excellent yields by the reaction of intermediate (**4**) with various R-X. To compare the biological activities of substituents of the NH group of piperazine ring, we prepared the title compounds by substitution of α-bromoacetophenone, benzyl bromide, alkyl bromide, and hetero aromatic bromide. Comparative data for novel hybrid compounds with respect to structures and yield are provided in Table 1. All of the synthesized compounds were characterized by ^1^H-NMR and ^13^C-NMR, and some representative compounds were characterized by high-resolution mass spectrometry (HRMS) analysis. The spectra of title compounds were in Supplementary Materials.

### 2.2. Biological Evaluation

#### 2.2.1. Anti-Inflammatory Activity

RAW 264.7 cells are widely used to establish inflammatory models in vitro. In this work, we investigated the anti-inflammatory activity of synthetic compounds in LPS-induced RAW 264.7 on the generation of NO. The results of the title hybrids are summarized in Table 2.

As shown in Table 2, the substituents of the NH group of the piperazine ring have an obvious influence on anti-inflammatory activities. To our delight, benzofuran–piperazine compounds **16**, **18**, and **22** displayed good anti-inflammatory activity on the generation of NO (IC_50_ < 10 μM). Especially, compound **16** was found to be the most potent anti-inflammatory agent (IC_50_ = 5.28 μM). In addition, compounds **5**, **9**, and **24** showed potent anti-inflammatory activity (IC_50_ < 20 μM). However, hetero aromatic compound **25** displayed no activity compared to others. Notably, except for the mentioned hybrids, most of the derivatives had little or no inhibitory effects on the release of NO (IC_50_ > 20 μM). On the other hand, we could find that the substituents of the NH group of the piperazine ring played an important role. Overall, keto- substituents contributed to better activity, alkyl and aryl substituents led to weaker activity, and the pyridyl substituent had no effect on anti-inflammatory activity (keto- > alkyl ≈ aryl > pyridyl). So, in future research, we will focus on the keto- substituents.

#### 2.2.2. Anticancer Activity

The anticancer activities of novel synthesized derivatives were evaluated against human lung cancer cell line (A549), human cervical carcinoma (Hela), and human gastric carcinoma (SGC7901) by MTT [3-(4, 5-dimethyl-2-thiazolyl)-2, 5-diphenyl-2-*H*-tetrazolium bromide] assay, using cisplatin (DDP) as the reference drug. The anti-tumor results for the hybrids are summarized in Table 3.

As shown in Table 3, the structures of the hybrid compounds have an obvious influence on cytotoxic activities. There were three series of substituents of the piperazine ring, including keto-, alkyl, and aryl. In general, derivatives bearing keto- substituent (**16**–**24**) were most active, displaying similar or better cytotoxic activity in vitro compared to cisplatin (DDP). However, hetero aromatic compound **25** had weak cytotoxic activity against Hela, and alkyl-substituted compounds showed no activity, except for hybrid **9** (IC_50_ = 19.27 μM against A549). Furthermore, the electron withdrawing group or halide substituent at position 4 of the benzene ring of the acetophenone moiety could be more sensitive to cytotoxic activity, such as 4-F (**19**), 4-Cl (**20**), and 4-CN (**23**). Compound **16** showed the most potent cytotoxic activity and pronounced selectivity against A549 (IC_50_ = 0.12 μM) and SGC7901 (IC_50_ = 2.75 μM). In addition, some aryl-substituted compounds displayed good inhibitory activity. For example, hybrids **11** and **12** had selective anti-tumor activity against cancer cells (IC_50_ = 8.57–16.27 μM).

**Scheme 4:**
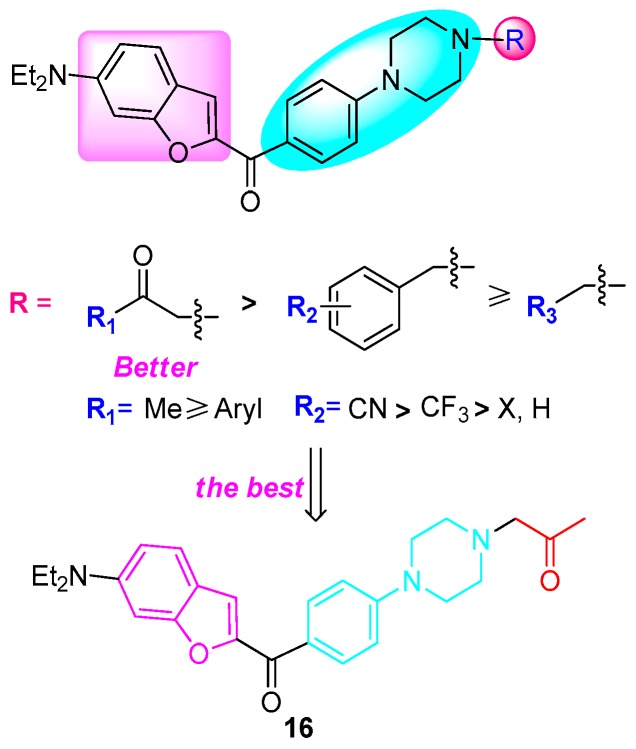
Structure–activity relationship of hybrid compounds.

The biological results suggested that the existence of a keto- substituent played an important role in the anti-inflammatory and anticancer activity of compounds. In all synthesized derivatives, hybrid **16** had better inhibitory effect on the generation of NO and showed more potent cytotoxic activity against A549 and SGC7901, and could be identified as the most potent anti-inflammatory and anti-tumor agent among those studied. The structure–activity relationship (SAR) results are summarized in Scheme 4.

Overall, although only a few compounds were found to exhibit excellent anti-inflammatory and antitumor activities, we could study the tendency of benzofuran–piperazine compounds and conduct further medicine chemistry research following the results.

## 3. Materials and Methods

### 3.1. General Information

Starting materials were commercially available and analytically pure. Melting points were measured on a YANACO microscopic melting point meter and were uncorrected. ^1^H-NMR and ^13^C-NMR spectra were recorded on Bruker AV 300 and Bruker AV 400 spectrometers (Bruker, Karlsruhe, Germany) using TMS as internal standard and CDCl_3_ as solvent, respectively. Thin layer chromatographic (TLC) analysis was carried out on silica gel plates GF_254_. High-resolution mass spectra were performed on an ESI Q-TOF MS spectrometer (Agilent, Santa Clara, CA, USA).

### 3.2. Chemistry

#### 3.2.1. General Procedure

General procedure for the synthesis of compound **3**: To a solution of acetone (50 mL), 5-diethylamino salicylaldehyde (1.93 g, 10 mmol) and 2-bromo-4′-fluoroacetophenone (2.17 g, 10 mmol), was added K_2_CO_3_ (2.76 g, 20 mmol) and left to react for 4 h in reflux. The reaction was poured into 100 mL cold water. After stirring for 10 min, the mixture was filtered, and the filtrate was concentrated in vacuo and dried to afford a yellow solid.

General procedure for the preparation of compound **4**: To a stirred solution of compound **3** (1.56 g, 5 mmol) and K_2_CO_3_ (1.38 g, 10 mmol) in dried DMF (20 mL), piperazine (0.86 g, 10 mmol) was added, and the reaction mixture was stirred for 12 h at 110 °C. After completion of the reaction as indicated by TLC, the reaction was quenched by the addition of CHCl_3_ (50 mL) and washed with water (3 × 20 mL). The organic layer was dried by anhydrous sodium sulfate, concentrated in vacuo, and purified by column chromatography to afford a brown solid.

General procedure for the preparation of hybrid derivatives **5**–**7**: To a stirred solution of compound **4** (0.5 mmol) in dried DMF (5 mL), NaH (0.06 g, 60% in oil) was added, and the mixture was stirred at 0 °C for 1 h. Then, R-X (1 mmol) was added, and after completion of the reaction as indicated by TLC, the reaction was quenched by the addition of H_2_O (30 mL) and was extracted with DCM (3 × 10 mL). The organic layer was dried by anhydrous sodium sulfate, concentrated in vacuo, and purified by column chromatography (1% MeOH/DCM) to afford products.

General procedure for the preparation of derivatives **8**–**14**, **25**: To a stirred solution of compound **4** (0.5 mmol) and Cs_2_CO_3_ (0.5 g) in dried DCM (15 mL), R-X (0.6 mmol) was added, and the reaction mixture was stirred for 12 h at room temperature (r.t.). After completion of the reaction as indicated by TLC, the mixture was filtered off. Then, the organic layer was concentrated in vacuo and purified by column chromatography (1% MeOH/DCM) to afford products.

General procedure for the preparation of derivatives **15**–**24**: To a stirred solution of compound **4** (0.5 mmol) and K_2_CO_3_ (0.2 g) in dried DCM (10 mL), R-X (0.6 mmol) was added, and the reaction mixture was stirred for 12 h at r.t. After completion of the reaction as indicated by TLC, the reaction was quenched by the addition of 5% NaOH (20 mL) and was extracted with DCM (3 × 10 mL). The organic layer was dried using anhydrous sodium sulfate, concentrated in vacuo, and purified by column chromatography (1% MeOH/DCM) to afford products.

#### 3.2.2. The Character of All Compounds

Compound **4**: Brown solid; ^1^H-NMR (300 MHz, CDCl_3_) δ: 8.02 (d, *J* = 9.0 Hz, 2H), 7.48 (d, *J* = 8.7 Hz, 1H), 7.38 (s, 1H), 6.95 (d, *J* = 9.0 Hz, 2H), 6.72–6.78 (m, 2H), 3.46 (q, *J* = 7.2 Hz, 4H), 3.35 (t, *J* = 4.8 Hz, 4H), 3.05 (t, *J* = 4.8 Hz, 4H), 1.90 (s, 1H), 1.23 (t, *J* = 6.9 Hz, 6H); ^13^C-NMR (75 MHz, CDCl_3_) δ: 181.94, 158.99, 154.37, 151.09, 149.06, 131.51, 128.06, 123.48, 116.76, 116.51, 113.71, 110.93, 93.16, 48.70, 45.99, 45.14, 12.63; HRMS (ESI-TOF): *m*/*z* calcd for C_23_H_27_N_3_O_2_Na [M + Na]^+^ 400.1995, found 400.1995.

Compound **5**: Pale yellow solid; ^1^H-NMR (400 MHz, CDCl_3_) δ: 8.02 (d, *J* = 8.9 Hz, 2H), 7.47 (d, *J* = 8.8 Hz, 1H), 7.37 (s, 1H), 6.94 (d, *J* = 8.9 Hz, 2H), 6.78 (s, 1H), 6.73–6.75 (dd, *J* = 2.2 Hz, 2.2 Hz, 1H), 3.37–3.43 (m, 8H), 2.58 (t, *J* = 5.2 Hz, 4H), 2.35 (s, 3H), 1.22 (t, *J* = 7.0 Hz, 6H); ^13^C-NMR (100 MHz, CDCl_3_) δ: 181.86, 158.99, 153.94, 151.22, 149.10, 131.53, 128.10, 123.45, 116.59, 113.75, 111.01, 93.27, 54.90, 47.56, 46.23, 45.14, 12.64.

Compound **6**: Yellow solid; ^1^H-NMR (400 MHz, CDCl_3_) δ: 8.01 (d, *J* = 9.0 Hz, 2H), 7.46 (d, *J* = 8.8 Hz, 1H), 7.37 (s, 1H), 6.93 (d, *J* = 9.0 Hz, 1H), 6.77 (s, 1H), 6.72–6.75 (dd, *J* = 2.2 Hz, 2.2 Hz, 1H), 3.38–3.44 (m, 8H), 2.63 (t, *J* = 5.1 Hz, 4H), 2.51 (q, *J* = 7.2 Hz, 2H), 1.22 (t, *J* = 7.0 Hz, 6H), 1.15 (t, *J* = 7.2 Hz, 3H); ^13^C-NMR (100 MHz, CDCl_3_) δ: 181.84, 158.95, 153.90, 151.11, 149.05, 131.48, 128.01, 123.43, 116.66, 116.50, 113.67, 110.95, 93.16, 52.55, 52.43, 47.46, 45.09, 12.60, 11.97.

Compound **7**: Pale brown solid; ^1^H-NMR (400 MHz, CDCl_3_) δ: 8.01 (d, *J* = 9.0 Hz, 2H), 7.45 (d, *J* = 8.8 Hz, 1H), 7.36 (s, 1H), 6.91 (d, *J* = 9.0 Hz, 2H), 6.76 (s, 1H), 6.71–6.73 (dd, *J* = 2.2 Hz, 2.2 Hz, 1H), 3.43 (q, *J* = 7.1 Hz, 4H), 3.37 (t, *J* = 5.2 Hz, 4H), 2.57 (t, *J* = 5.0 Hz, 4H), 2.538 (t, *J* = 7.6 Hz, 2H), 1.25–1.30 (m, 32H), 1.20 (t, *J* = 7.0 Hz, 6H), 0.88 (t, *J* = 6.6 Hz, 3H); ^13^C-NMR (100 MHz, CDCl_3_) δ: 181.71, 158.90, 153.96, 151.20, 149.00, 131.46, 127.86, 123.37, 116.53, 116.44, 113.53, 110.92, 93.21, 58.83, 53.03, 47.52, 45.06, 31.99, 29.76, 29.69, 29.67, 29.42, 27.64, 26.95, 22.75, 14.17, 12.58; HRMS (ESI-TOF): *m*/*z* calcd for C_41_H_63_N_3_O_2_ [M + H]^+^ 630.4993, found 630.4983.

Compound **8**: Yellow solid; ^1^H-NMR (300 MHz, CDCl_3_) δ: 8.02 (d, *J* = 8.7 Hz, 2H), 7.48 (d, *J* = 8.7 Hz, 1H), 7.38 (s, 1H), 6.95 (d, *J* = 8.7 Hz, 2H), 6.72–6.78 (m, 2H), 5.83–5.97 (m, 1H), 5.26 (d, *J* = 15.0 Hz, 1H), 5.21 (d, *J* = 8.1 Hz, 1H), 3.37–3.46 (m, 8H), 3.08 (d, *J* = 6.6 Hz, 2H), 2.63 (t, *J* = 5.1 Hz, 4H), 1.23 (t, *J* = 6.9 Hz, 6H); ^13^C-NMR (75 MHz, CDCl_3_) δ:181.94, 158.98, 153.98, 151.11, 149.05, 134.75, 131.53, 128.03, 123.47, 118.60, 116.74, 116.51, 113.70, 110.93, 93.17, 61.86, 52.85, 47.57, 45.14, 12.63; HRMS (ESI-TOF): *m*/*z* calcd for C_26_H_31_N_3_O_2_Na [M + Na]^+^ 440.2308, found 440.2307.

Compound **9**: Pale yellow solid; ^1^H-NMR (400 MHz, CDCl_3_) δ: 8.02 (d, *J* = 9.0 Hz, 2H), 7.47 (d, *J* = 8.8 Hz, 1H), 7.37 (s, 1H), 6.94 (d, *J* = 9.0 Hz, 2H), 6.77 (s, 1H), 6.72–6.75 (dd, *J* = 2.2 Hz, 2.2 Hz, 1H), 3.36–3.43 (m, 10H), 2.73 (t, *J* = 5.1 Hz, 4H), 2.28 (s, 1H), 1.22 (t, *J* = 7.1 Hz, 6H); ^13^C-NMR (100 MHz, CDCl_3_) δ: 181.81, 158.97, 153.86, 151.19, 149.09, 131.50, 128.16, 123.43, 116.60, 116.55, 113.80, 110.99, 93.23, 78.47, 73.65, 51.62, 47.54, 47.00, 45.11, 12.62; HRMS (ESI-TOF): *m*/*z* calcd for C_26_H_29_N_3_O_2_ [M + H]^+^ 416.2333, found 416.2337.

Compound **10**: Yellow solid; ^1^H-NMR (400 MHz, CDCl_3_) δ: 8.01 (d, *J* = 8.3 Hz, 2H), 7.46 (d, *J* = 8.8 Hz, 1H), 7.25–7.37 (m, 6H), 6.91 (d, *J* = 8.5 Hz, 2H), 6.78 (s, 1H), 6.74 (d, *J* = 6.7 Hz, 1H), 3.55 (s, 2H), 3.43 (q, *J* = 8.8 Hz, 4H), 3.36 (t, *J* = 4.7 Hz, 4H), 2.60 (t, *J* = 4.4 Hz, 4H), 1.21 (t, *J* = 6.9 Hz, 6H); ^13^C-NMR (100 MHz, CDCl_3_) δ: 181.79, 158.91, 153.97, 151.12, 149.00, 137.88, 131.47, 129.23, 128.39, 127.84, 127.29, 123.41, 116.58, 116.49, 113.57, 110.91, 93.14, 63.07, 52.81, 47.51, 45.07, 12.59.

Compound **11**: Pale red solid; ^1^H-NMR (300 MHz, CDCl_3_) δ: 8.00 (d, *J* = 8.7 Hz, 2H), 7.62 (d, *J* = 8.1 Hz, 2H), 7.44–7.48 (m, 3H), 7.36 (s, 1H), 6.92 (d, *J* = 9.0 Hz, 2H), 6.76 (s, 1H), 6.71–6.76 (dd, *J* = 2.1 Hz, 2.1 Hz, 1H), 3.58 (s, 2H), 3.33–3.44 (m, 8H), 2.59 (t, *J* = 4.8 Hz, 4H), 1.22 (t, *J* = 6.9 Hz, 6H); ^13^C-NMR (75 MHz, CDCl_3_) δ: 181.78, 158.93, 153.83, 150.99, 149.01, 143.91, 132.25, 131.45, 129.56, 128.04, 123.44, 119.00, 116.73, 116.40, 113.68, 111.05, 93.02, 62.39, 52.86, 47.51, 45.07, 12.57.

Compound **12**: Brown solid; ^1^H-NMR (400 MHz, CDCl_3_) δ: 8.02 (d, *J* = 9.0 Hz, 2H), 7.59 (d, *J* = 8.1 Hz, 2H), 7.48 (d, *J* = 3.1 Hz, 2H), 7.46 (d, *J* = 3.9 Hz, 1H), 7.38 (s, 1H), 6.91 (d, *J* = 9.0 Hz, 2H), 6.78 (s, 1H), 6.72–6.75 (dd, *J* = 2.2 Hz, 2.2 Hz, 1H), 3.58 (s, 2H), 3.43 (q, *J* = 7.0 Hz, 4H), 3.36 (t, *J* = 4.9 Hz, 4H), 2.58 (t, *J* = 5.0 Hz, 4H), 1.21 (t, *J* = 7.0 Hz, 6H); ^13^C-NMR (100 MHz, CDCl_3_) δ: 181.68, 158.90, 153.87, 151.08, 149.01, 142.32, 131.43, 129.24, 127.92, 125.32, 125.29, 125.25, 125.22, 123.40, 116.57, 116.44, 113.60, 110.91, 93.06, 62.34, 52.78, 47.46, 45.02, 12.53; HRMS (ESI-TOF): *m*/*z* calcd for C_31_H_32_F_3_N_3_O_2_ [M + H]^+^ 536.2519, found 536.2526.

Compound **13**: Pale yellow solid; ^1^H-NMR (400 MHz, CDCl_3_) δ: 8.01 (d, *J* = 9.0 Hz, 2H), 7.46 (d, *J* = 8.8 Hz, 1H), 7.37 (s, 1H), 7.19- 7.22 (m, 1H), 7.03–7.17 (m, 2H), 8.01 (d, *J* = 9.0 Hz, 2H), 6.77 (s, 1H), 6.72–6.74 (dd, *J* = 2.2 Hz, 2.2 Hz, 1H), 3.46 (s, 2H), 3.43 (q, *J* = 7.1 Hz, 4H), 3.35 (t, *J* = 4.9 Hz, 4H), 2.56 (t, *J* = 5.0 Hz, 4H), 1.21 (t, *J* = 7.1 Hz, 6H); ^13^C-NMR (100 MHz, CDCl_3_) δ: 181.67, 158.88, 153.84, 151.05, 148.99, 135.27, 135.22, 135.18, 131.41, 127.89, 124.76, 124.72, 124.70, 124.66, 123.39, 117.69, 117.52, 117.02, 116.85, 116.57, 116.42, 113.58, 110.90, 93.04, 61.76, 52.66, 47.45, 45.01, 12.53; HRMS (ESI-TOF): *m*/*z* calcd for C_30_H_32_F_2_N_3_O_2_ [M + H]^+^ 504.2457, found 504.2453.

Compound **14**: Pale brown solid; ^1^H-NMR (400 MHz, CDCl_3_) δ: 8.20 (d, *J* = 8.8 Hz, 2H), 8.02 (d, *J* = 9.0 Hz, 2H), 7.55 (d, *J* = 8.8 Hz, 2H), 7.47 (d, *J* = 8.8 Hz, 1H), 7.37 (s, 1H), 6.93 (d, *J* = 9.0 Hz, 2H), 6.77 (s, 1H), 6.73–6.76 (dd, *J* = 2.2 Hz, 2.2 Hz, 1H), 3.64 (s, 2H), 3.36–3.45 (m, 8H), 2.62 (t, *J* = 5.1 Hz, 4H), 1.23 (t, *J* = 7.0 Hz, 6H); ^13^C-NMR (100 MHz, CDCl_3_) δ: 181.79, 158.99, 153.88, 151.18, 149.13, 147.41, 146.08, 131.52, 129.59, 128.20, 123.71, 123.45, 116.63, 116.54, 113.77, 111.03, 93.21, 62.17, 52.97, 47.63, 45.12, 12.63.

Compound **15**: Yellow solid; ^1^H-NMR (300 MHz, CDCl_3_) δ: 8.01 (d, *J* = 8.7 Hz, 2H), 7.47 (d, *J* = 8.7 Hz, 1H), 7.37 (s, 1H), 6.94 (d, *J* = 8.7 Hz, 2H), 6.77 (s, 1H), 6.75 (d, *J* = 9.0 Hz, 1H), 4.24 (q, *J* = 6.9 Hz, 2H), 3.45 (q, *J* = 6.9 Hz, 8H), 3.27 (s, 2H), 2.76 (t, *J* = 5.1 Hz, 4H), 1.31 (t, *J* = 7.2 Hz, 3H), 1.22 (t, *J* = 7.2 Hz, 6H); ^13^C-NMR (75 MHz, CDCl_3_) δ: 181.91, 170.21, 158.97, 153.85, 151.06, 149.04, 131.50, 128.15, 123.47, 116.77, 113.79, 110.92, 93.12, 60.88, 59.46, 52.75, 47.47, 45.12, 14.37, 12.61; HRMS (ESI-TOF): *m*/*z* calcd for C_27_H_34_N_3_O_4_ [M + H]^+^ 464.2543, found 464.2545.

Compound **16**: Pale red solid; ^1^H-NMR (400 MHz, CDCl_3_) δ: 7.99 (d, *J* = 8.9 Hz, 2H), 7.45 (d, *J* = 8.8 Hz, 1H), 7.35 (s, 1H), 6.90 (d, *J* = 9.0 Hz, 2H), 6.75 (s, 1H), 6.70–6.73 (dd, *J* = 2.2 Hz, 2.2 Hz, 1H), 3.36–3.40 (m, 8H), 3.23 (s, 2H), 2.62 (t, *J* = 5.0 Hz, 4H), 2.14 (s, 3H), 1.19 (t, *J* = 7.0 Hz, 6H); ^13^C-NMR (100 MHz, CDCl_3_) δ: 206.03, 181.69, 158.87, 153.75, 151.00, 148.98, 131.39, 128.00, 123.38, 116.60, 116.39, 113.65, 110.89, 93.02, 67.93, 52.98, 47.33, 45.00, 27.81, 12.52; HRMS (ESI-TOF): *m/z* calcd for C_26_H_31_N_3_O_3_ [M + H]^+^ 434.2438, found 434.2444.

Compound **17**: Pale yellow solid; ^1^H-NMR (300 MHz, CDCl_3_) δ: 8.03 (d, *J* = 9.0 Hz, 4H), 7.59 (d, *J* = 7.2 Hz, 1H), 7.45–7.50 (m, 3H), 7.38 (s, 1H), 6.96 (d, *J* = 9.0 Hz, 2H), 6.78 (s, 1H), 6.73–6.77 (dd, *J* = 2.1 Hz, 2.1 Hz, 1H), 3.90 (s, 2H), 3.39–3.48 (m, 8H), 2.80 (t, *J* = 4.8 Hz, 4H), 1.24 (t, *J* = 7.2 Hz, 6H); ^13^C-NMR (75 MHz, CDCl_3_) δ: 196.18, 181.89, 158.96, 153.88, 151.10, 149.05, 136.01, 133.52, 131.50, 128.73, 128.20, 123.45, 116.72, 116.50, 113.76, 110.94, 93.16, 64.36, 53.26, 47.50, 45.10, 12.61; HRMS (ESI-TOF): *m*/*z* calcd for C_31_H_34_N_3_O_3_ [M + H]^+^ 496.2595, found 496.2598.

Compound **18**: Pale red solid; ^1^H-NMR (300 MHz, CDCl_3_) δ: 8.59 (s, 1H), 7.97–8.09 (m, 4H), 7.93 (t, *J* = 8.7 Hz, 2H), 7.54–7.64 (m, 2H), 7.49 (d, *J* = 8.7 Hz, 1H), 7.39 (s, 1H), 6.97 (d, *J* = 8.7 Hz, 2H), 6.78 (s, 1H), 6.73–6.77 (dd, *J* = 2.1 Hz, 2.1 Hz, 1H), 4.04 (s, 2H), 3.40–3.50 (m, 8H), 2.85 (t, *J* = 5.1 Hz, 4H), 1.24 (t, *J* = 6.9 Hz, 6H); ^13^C-NMR (75 MHz, CDCl_3_) δ: 196.21, 182.00, 159.02, 153.94, 149.11, 135.87, 132.60, 131.56, 129.96, 129.76, 128.80, 128.65, 128.21, 127.97, 127.03, 123.96, 123.49, 116.75, 116.57, 113.83, 110.99, 93.24, 64.56, 53.38, 47.60, 45.16, 12.66; HRMS (ESI-TOF): *m*/*z* calcd for C_35_H_35_N_3_O_3_Na [M + Na]^+^ 568.2570, found 568.2569.

Compound **19**: Orange solid; ^1^H-NMR (300 MHz, CDCl_3_) δ: 8.04–8.08 (m, 2H), 8.01 (d, *J* = 8.7 Hz, 2H), 7.47 (d, *J* = 8.7 Hz, 1H), 7.37 (s, 1H), 7.16 (t, *J* = 8.4 Hz, 2H), 6.94 (d, *J* = 8.7 Hz, 2H), 6.77 (s, 1H), 6.72–6.75 (dd, *J* = 2.1 Hz, 2.1 Hz, 1H), 3.84 (s, 2H), 3.38–3.45 (m, 8H), 2.77 (t, *J* = 5.1 Hz, 4H), 1.22 (t, *J* = 6.9 Hz, 6H); ^13^C-NMR (75 MHz, CDCl_3_) δ: 194.77, 181.92, 159.00, 153.86, 151.05, 149.08, 132.41, 131.51, 131.10, 130.97, 128.19, 123.49, 116.82, 116.49, 116.01, 115.72, 113.81, 110.95, 93.12, 64.51, 53.26, 47.51, 45.12, 12.62.

Compound **20**: Pale brown solid; ^1^H-NMR (300 MHz, CDCl_3_) δ: 8.01 (d, *J* = 9.3 Hz, 2H), 7.98 (d, *J* = 9.0 Hz, 2H), 7.47 (d, *J* = 8.4 Hz, 1H), 7.42 (d, *J* = 8.4 Hz, 2H), 7.37 (s, 1H), 6.94 (d, *J* = 9.0 Hz, 2H), 6.77 (s, 1H), 6.72–6.77 (dd, *J* = 2.1 Hz, 2.1 Hz, 1H), 3.83 (s, 2H), 3.43 (t, *J* = 7.2 Hz, 8H), 2.76 (t, *J* = 4.8 Hz, 4H), 1.22 (t, *J* = 7.2 Hz, 6H); ^13^C-NMR (75 MHz, CDCl_3_) δ: 195.17, 181.86, 158.96, 153.82, 151.01, 149.04, 139.94, 134.21, 131.48, 129.75, 129.03, 128.15, 123.47, 116.79, 116.44, 113.78, 110.92, 93.07, 64.50, 53.22, 47.47, 45.10, 12.60; HRMS (ESI-TOF): *m*/*z* calcd for C_31_H_32_N_3_O_3_NaCl [M + Na]^+^ 552.2024, found 552.2023.

Compound **21**: Pale green solid; ^1^H-NMR (300 MHz, CDCl_3_) δ: 8.03 (d, *J* = 8.7 Hz, 2H), 7.92 (d, *J* = 8.4 Hz, 2H), 7.63 (d, *J* = 8.7 Hz, 2H), 7.48 (d, *J* = 8.7 Hz, 1H), 7.38 (s, 1H), 6.96 (d, *J* = 9.0 Hz, 2H), 6.78 (s, 1H), 6.73–6.77 (dd, *J* = 2.1 Hz, 2.1 Hz, 1H), 3.84 (s, 2H), 3.44 (t, *J* = 7.2 Hz, 8H), 2.78 (t, *J* = 5.1 Hz, 4H), 1.24 (t, *J* = 6.9 Hz, 6H); HRMS (ESI-TOF): *m*/*z* calcd for C_31_H_32_BrN_3_O_3_ [M + H]^+^ 574.1700, found 574.1699.

Compound **22**: Orange solid; ^1^H-NMR (400 MHz, CDCl_3_) δ: 8.00 (d, *J* = 8.9 Hz, 2H), 7.99 (d, *J* = 8.8 Hz, 2H), 7.45 (d, *J* = 8.8 Hz, 2H), 7.36 (s, 1H), 6.92 (d, *J* = 8.8 Hz, 2H), 6.75 (s, 1H), 6.73 (d, *J* = 8.8 Hz, 1H), 3.83 (s, 3H), 3.82 (s, 2H), 3.37–3.43 (m, 8H), 2.76 (t, *J* = 5.0 Hz, 4H), 1.20 (t, *J* = 7.0 Hz, 6H); ^13^C-NMR (100 MHz, CDCl_3_) δ: 194.62, 181.78, 163.78, 158.92, 153.85, 151.08, 149.05, 131.46, 130.52, 129.05, 128.00, 123.42, 116.66, 116.48, 113.83, 113.69, 110.96, 93.12, 64.02, 55.53, 53.14, 47.37, 45.05, 12.57; HRMS (ESI-TOF): *m*/*z* calcd for C_32_H_36_N_3_O_4_ [(M + H)]^+^ 526.2700, found 526.2700.

Compound **23**: Pale yellow solid; ^1^H-NMR (400 MHz, CDCl_3_) δ: 8.11 (d, *J* = 8.4 Hz, 2H), 8.00 (d, *J* = 8.9 Hz, 2H), 7.76 (d, *J* = 8.4 Hz, 2H), 7.46 (d, *J* = 8.8 Hz, 1H), 7.36 (s, 1H), 6.92 (d, *J* = 9.0 Hz, 2H), 6.72–6.75 (m, 2H), 3.86 (s, 2H), 3.44 (q, *J* = 7.1 Hz, 8H), 2.76 (t, *J* = 4.9 Hz, 4H), 1.21 (t, *J* = 7.0 Hz, 6H); ^13^C-NMR (100 MHz, CDCl_3_) δ: 195.18, 181.85, 159.01, 153.77, 151.06, 149.15, 138.84, 132.57, 131.51, 128.84, 128.30, 123.49, 117.96, 116.83, 116.74, 116.50, 113.87, 111.05, 93.14, 64.70, 53.15, 47.48, 45.11, 12.61; HRMS (ESI-TOF): *m*/*z* calcd for C_32_H_33_N_4_O_3_ [M + H]^+^ 521.2547, found 521.2547.

Compound **24**: Pale green solid; ^1^H-NMR (400 MHz, CDCl_3_) δ: 8.04 (q, *J* = 8.6 Hz, 2H), 7.93 (q, *J* = 7.9 Hz, 2H), 7.49 (q, *J* = 8.7 Hz, 1H), 7.40 (d, *J* = 15.3 Hz, 1H), 7.28 (q, *J* = 7.7 Hz, 2H), 6.95 (q, *J* = 8.6 Hz, 2H), 6.79 (d, *J* = 13.3 Hz, 1H), 6.73 (d, *J* = 8.9 Hz, 1H), 3.88 (d, *J* = 15.4 Hz, 2H), 3.39–3.44 (m, 8H), 2.79 (t, *J* = 4.1 Hz, 4H), 2.42 (t, *J* = 6.2 Hz, 3H), 1.16–1.24 (m, 6H); ^13^C-NMR (100 MHz, CDCl_3_) δ: 195.75, 181.80, 158.95, 153.88, 151.15, 149.08, 144.33, 133.55, 131.49, 129.37, 128.30, 128.05, 123.42, 116.62, 116.52, 113.72, 110.98, 93.17, 64.15, 53.17, 47.43, 45.07, 21.74, 12.59.

Compound **25**: Brown solid; ^1^H-NMR (400 MHz, CDCl_3_) δ: 8.56 (d, *J* = 6.0 Hz, 2H), 8.01 (d, *J* = 8.9 Hz, 2H), 7.46 (d, *J* = 8.8 Hz, 1H), 7.37 (s, 1H), 7.31 (d, *J* = 5.9 Hz, 1H), 6.93 (d, *J* = 9.0 Hz, 2H), 6.77 (s, 1H), 6.75 (d, *J* = 8.8 Hz, 1H), 3.56 (s, 2H), 3.36–3.44 (m, 8H), 2.61 (t, *J* = 5.0 Hz, 4H), 1.22 (t, *J* = 7.0 Hz, 6H); ^13^C-NMR (100 MHz, CDCl_3_) δ: 181.81, 158.98, 153.89, 151.15, 150.57, 150.01, 149.09, 147.36, 131.50, 128.15, 123.92, 123.44, 121.35, 116.64, 116.52, 114.50, 113.74, 110.98, 93.19, 61.76, 52.97, 47.60, 45.11, 12.62; HRMS (ESI-TOF): *m*/*z* calcd for C_29_H_33_N_4_O_2_ [M + H]^+^ 469.2598, found 469.2601.

### 3.3. Biological Activity Experiments

#### 3.3.1. Anti-Inflammatory Activity

Murine RAW264.7 macrophages were plated in 96-well plate at a density of 1 × 10^5^ cells/well and stimulated with 1 μg/mL LPS in the presence or absence of various concentrations of compound for 24 h. The production of NO was determined by assaying culture supernatant for NO^2−^. Supernatant (100 μL) was mixed with an equal volume of Griess reagent at r.t. for 10 min. Absorbance was measured at 540 nm in a microplate reader.

#### 3.3.2. Antitumor Activity

The assay was carried out using the method described previously. About 1 × 10^4^ cell/well were seeded into 96-well microtiter plates. At twenty-four hours post-seeding, cells were treated with vehicle control or various concentrations of samples for 48 h. Twenty microliters of MTT solution (5 mg/mL) was added to each well, and the tumor cells were incubated at 37 °C in a humidified atmosphere of 5% CO_2_ air for 4 h. Upon removal of MTT/medium, 150 μL of DMSO was added to each well, and the plate was agitated at oscillator for 5 min to dissolve the MTT-formazan. The assay plate was read at a wavelength of 570 nm using a microplate reader.

## 4. Conclusions

In summary, a series of novel hybrid compounds between benzofuran and *N*-aryl piperazine have been synthesized and screened in vitro for anti-inflammatory and anticancer activity. The results demonstrated that derivative **16** not only had an inhibitory effect on the generation of NO (IC_50_ = 5.28 μM), but also displayed good cytotoxic activity against A549 and SGC7901 (IC_50_ = 0.12 μM and 2.75 μM, respectively), which was considered to be the most potent anti-inflammatory and anti-tumor agent in this study. Further research is currently underway, and the results will be reported in due course.

## Figures and Tables

**Table 1:**
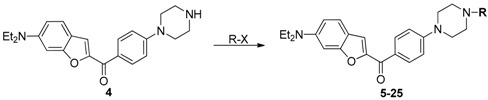
Structures and yields of compounds **5**–**25**.

Compound	R	m.p. (°C)	Yields (%) ^a^
**5**	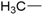	171–173	51
**6**	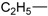	171–173	57
**7**	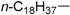	185–186	68
**8**	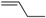	174–176	83
**9**	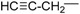	176–178	81
**10**	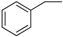	179–181	83
**11**	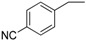	186–188	79
**12**	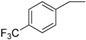	180–182	76
**13**	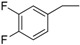	178–180	82
**14**	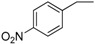	187–189	81
**15**	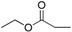	156–158	91
**16**	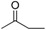	161–163	86
**17**	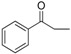	188–190	82
**18**	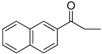	202–204	70
**19**	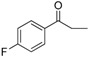	192–193	84
**20**	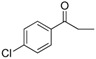	194–196	75
**21**	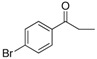	198–200	83
**22**	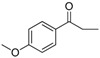	181–183	85
**23**	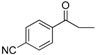	204–206	77
**24**	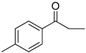	184–186	82
**25**	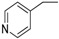	135–137	86

^a^ Yields represent isolated yields.

**Table 2:** Anti-inflammatory activities of compounds.

Compound	NO Generation (IC_50_, μM) ^a^	Compound	NO Generation (IC_50_, μM) ^a^
**5**	14.12	**16**	5.28
**6**	34.24	**17**	25.40
**7**	>40	**18**	6.53
**8**	>40	**19**	>40
**9**	18.52	**20**	>40
**10**	>40	**21**	>40
**11**	>40	**22**	9.13
**12**	>40	**23**	23.56
**13**	23.06	**24**	18.37
**14**	20.27	**25**	>40
**15**	31.68		

^a^ Values represent the concentration required to produce 50% inhibition of the response.

**Table 3:** In vitro cytotoxic activities of title compounds.

Compound	Cell Lines (IC_50_, μM) ^a^		
	A549	Hela	SGC7901
**5**	>40	>40	27.24
**6**	>40	32.53	>40
**7**	>40	>40	>40
**8**	>40	>40	>40
**9**	19.27	>40	>40
**10**	>40	>40	>40
**11**	16.14	8.57	>40
**12**	>40	25.14	16.27
**13**	>40	>40	25.04
**14**	>40	33.24	>40
**15**	>40	22.36	30.43
**16**	0.12	26.32	2.75
**17**	27.82	>40	15.41
**18**	19.34	>40	23.92
**19**	6.25	18.71	36.23
**20**	8.11	28.74	>40
**21**	23.22	15.35	>40
**22**	26.07	>40	>40
**23**	34.13	12.68	7.45
**24**	>40	27.58	>40
**25**	>40	26.22	>40
**DDP**	11.54	20.52	12.44

^a^ Cytotoxicity as IC_50_ values for each cell line, the concentration of compound that inhibits 50% of the cell growth measured by MTT assay. DDP: cisplatin.

## Supplementary Materials

The ^1^H-NMR, ^13^C-NMR and HRMS spectra are available online at: http://www.mdpi.com/1420-3049/21/12/1684/s1.

## References

[1] Xue S.T., Guo H.F., Liu M.J., Jin J., Ju D.H., Liu Z.Y., Li Z.R. (2015). Synthesis of a novel class of substituted benzothiophene or benzofuran derivatives as BMP-2 up-regulators and evaluation of the BMP-2-up-regulating effects in vitro and the effects on glucocorticoid-induced osteoporosis in rats. Eur. J. Med. Chem..

[2] Hassan G.S., Abou-Seri S.M., Kamel G., Ali M.M. (2014). Celecoxib analogs bearing benzofuran moiety as cyclooxygenase-2 inhibitors: Design, synthesis and evaluation as potential anti-inflammatory agents. Eur. J. Med. Chem..

[3] Carrër A., Brinet D., Florent J.C., Rousselle P., Bertounesque E. (2012). Palladium-Catalyzed Direct Arylation of Polysubstituted Benzofurans. J. Org. Chem..

[4] Meshram H.M., Reddy B.C., Prasad B.R.V., Goud P.R., Kumar G.S., Kumar R.N. (2012). DABCO-Promoted Efficient and Convenient Synthesis of Benzofurans. Synth. Commun..

[5] Kumaraswamy G., Ramakrishna G., Raju R., Padmaja M. (2010). An expedient synthesis of enantioenriched substituted (2-benzofuryl)arylcarbinols via tandem Rap–Stoermer and asymmetric transfer hydrogenation reactions. Tetrahedron.

[6] Watanabe H., Kawasaki A., Sano K., Ono M., Saji H. (2016). Synthesis and evaluation of copper-64 labeled benzofuran derivatives targeting β-amyloid aggregates. Bioorg. Med. Chem..

[7] Na M., Hoang D.M., Njamen D., Mbafor J.T., Fomum Z.T., Thuong P.T., Ahn J.S., Oh W.K. (2007). Inhibitory effect of 2-arylbenzofurans from Erythrina addisoniae on protein tyrosine phosphatase-1B. Bioorg. Med. Chem. Lett..

[8] Tian Y., Liang Z., Xu H., Mou Y., Guo C. (2016). Design, Synthesis and Cytotoxicity of Novel Dihydroartemisinin-Coumarin Hybrids via Click Chemistry. Molecules.

[9] Liang Z., Xu H., Tian Y., Guo M., Su X., Guo C. (2016). Design, Synthesis and Antifungal Activity of Novel Benzofuran-Triazole Hybrids. Molecules.

[10] Wang X.Q., Liu L.X., Li Y., Sun C.J., Chen W., Li L., Zhang H.B., Yang X.D. (2013). Design, synthesis and biological evaluation of novel hybrid compounds of imidazole scaffold-based 2-benzylbenzofuran as potent anticancer agents. Eur. J. Med. Chem..

[11] Vinh T.K., Yee S.W., Kirby1 A.J., Nicholls1 P.J., Simons C. (2001). 1-[(Benzofuran-2-yl) phenylmethyl] triazoles as steroidogenic inhibitors: Synthesis and in vitro inhibition of human placental CYP19 aromatase. Anti-Cancer Drug Des..

[12] Romagnoli R., Baraldi P.G., Sarkar T., Carrion M.D., Cruz-Lopez O., Cara C.L., Tolomeo M., Grimaudo S., Cristina A.D., Pipitone M.R. (2008). Synthesis and biological evaluation of 2-(3′,4′,5-trimethoxy benzoyl)-3-*N*,*N*-dimethylamino benzo[b]furan derivatives as inhibitors of tubulin polymerization. Bioorg. Med. Chem..

[13] Pevet I., Brulé C., Tizot A., Gohier A., Cruzalegui F., Boutin J.A., Goldstein S. (2011). Synthesis and pharmacological evaluation of thieno[2,3-*b*]pyridine derivatives as novel c-Src inhibitors. Bioorg. Med. Chem..

[14] Yang X.D., Wan W.C., Deng X.Y., Li Y., Yang L.J., Li L., Zhang H.B. (2012). Design, synthesis and cytotoxic activities of novel hybrid compounds between 2-phenylbenzofuran and imidazole. Bioorg. Med. Chem. Lett..

[15] Mao Z.W., Wan C.P., Jiang Y., Guo W.L., Rao G.X. (2015). Synthesis and Anti-tumor activity in vitro of *N*-hetercycle substitued benzofuran derivatives. J. China Pharm. Univ..

[16] Vergelli C., Ciciani G., Cilibrizzi A., Crocetti L., Mannelli L.D.C., Ghelardini C., Guerrini G., Iacovone A., Giovannoni M.P. (2015). Synthesis of five and six-membered heterocycles bearing an arylpiperazinylalkyl side chain as orally active antinociceptive agents. Bioorg. Med. Chem..

[17] Biswas N.N., Kutty S.K., Iskander G.M., Mielczarek M., Bhadbhade M.M., Gardner C.R., Black D.S., Kumar N. (2016). Synthesis of brominated novel *N*-heterocycles: New scaffolds for antimicrobial discovery. Tetrahedron.

[18] Chaudhary P., Kumar R., Verma A.K., Singh D., Yadav V., Chhillar A.K., Sharma G.L., Chandra R. (2006). Synthesis and antimicrobial activity of *N*-alkyl and *N*-aryl piperazine derivatives. Bioorg. Med. Chem..

[19] Zhao H.Y., Prosser A.R., Liottaa D.C., Wilson L.J. (2015). Discovery of novel *N*-aryl piperazine CXCR4 antagonists. Bioorg. Med. Chem. Lett..

[20] Mao Z.W., Zheng X., Lin Y.P., Hu C.Y., Wang X.L., Wan C.P., Rao G.X. (2016). Design, synthesis and anticancer activity of novel hybrid compounds between benzofuran and *N*-aryl piperazine. Bioorg. Med. Chem. Lett..

[21] Mao Z.W., Zheng X., Qi Y., Zhang M.D., Huang Y., Wan C.P., Rao G.X. (2016). Synthesis and biological evaluation of novel hybrid compounds between chalcone and piperazine as potential antitumor agents. RSC Adv..

[22] Mao Z.W., Zheng X., Lin Y.P., Qi Y., Hu C.Y., Wan C.P., Rao G.X. (2016). Concise synthesis and biological evaluation of chalcone derivatives bearing *N*-heterocyclic moieties. Heterocycles.

