# Supplementary material for: Design, Synthesis, and Biological Evaluation of Novel Benzofuran Derivatives Bearing N-Aryl Piperazine Moiety
Source: Molecules. 2016 Dec 9;21(12):1684. doi: 10.3390/molecules21121684 (PMC6274084; doi:10.3390/molecules21121684)
Supplement: Supplementary file 1 [file molecules-21-01684-s001.pdf]

Supplementary Materials: Design, Synthesis and Biological Evaluation of Novel Benzofuran Derivatives Bearing *N*-Aryl Piperazine Moiety

Yulu Ma, Xi Zheng, Hui Gao, Chunping Wan, Gaoxiong Rao and Zewei Mao

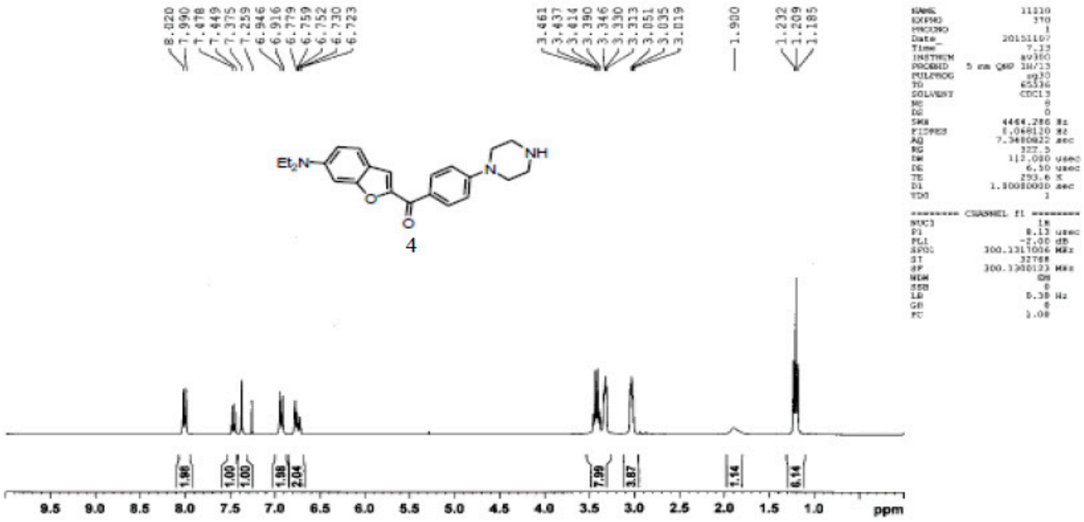

Figure S1. ^{1}H-NMR spectrum of compound 4.

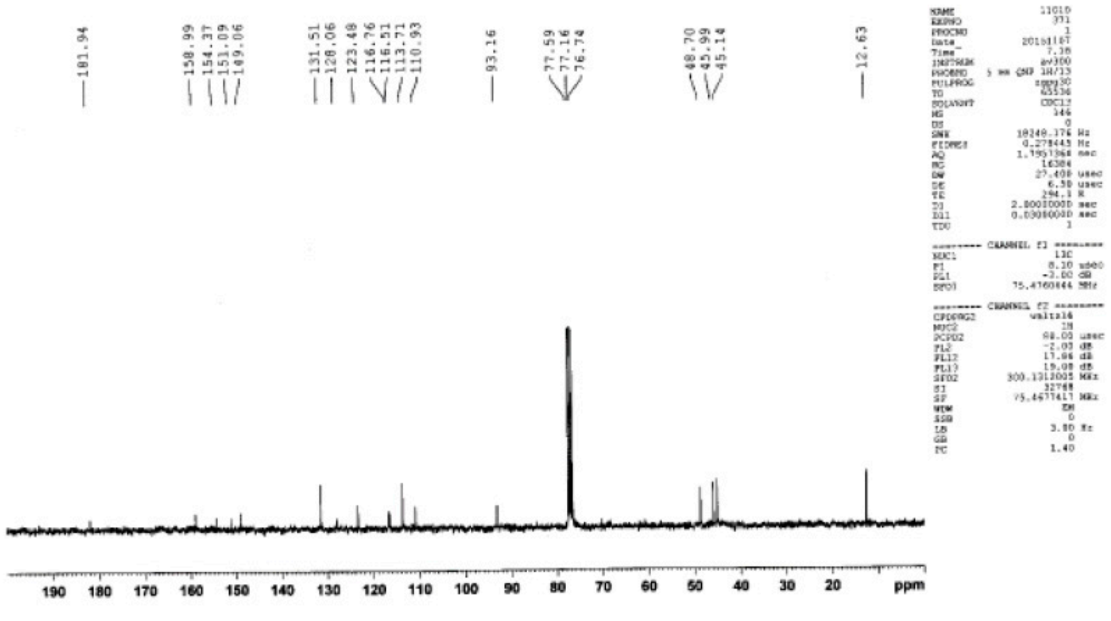

Figure S2. ^{13}C-NMR spectrum of compound 4.

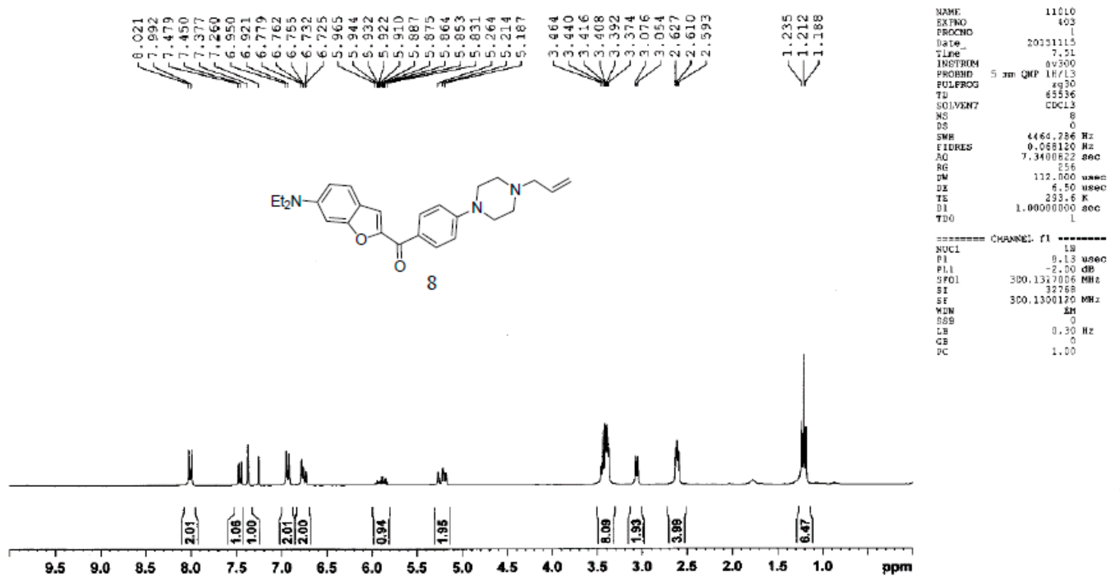

Figure S3. ^{1}H-NMR spectrum of compound 8.

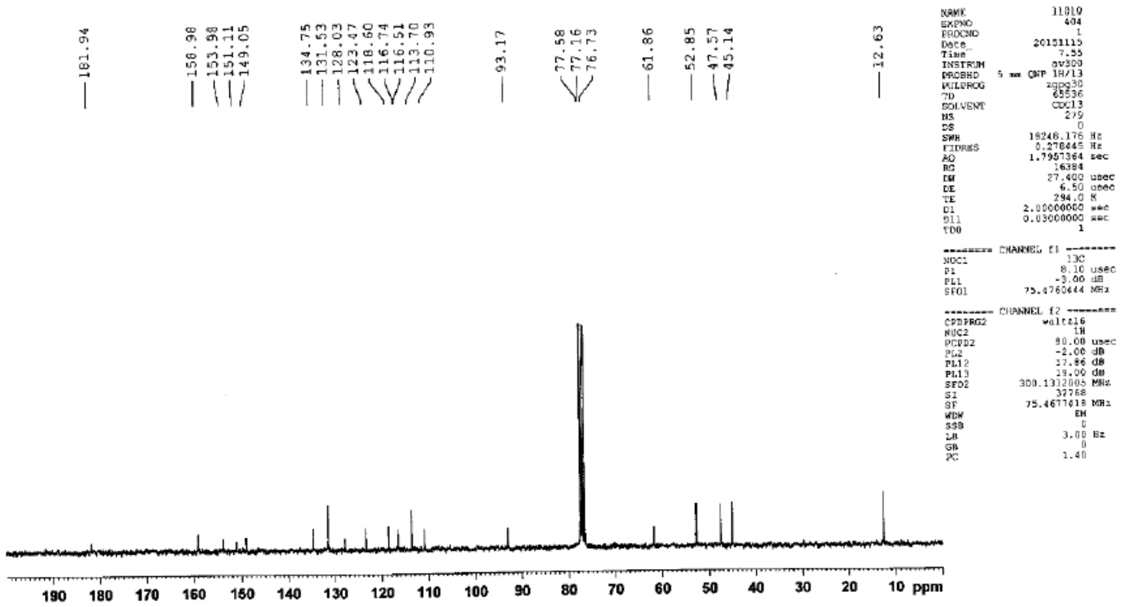

Figure S4. ^{13}C-NMR spectrum of compound 8.

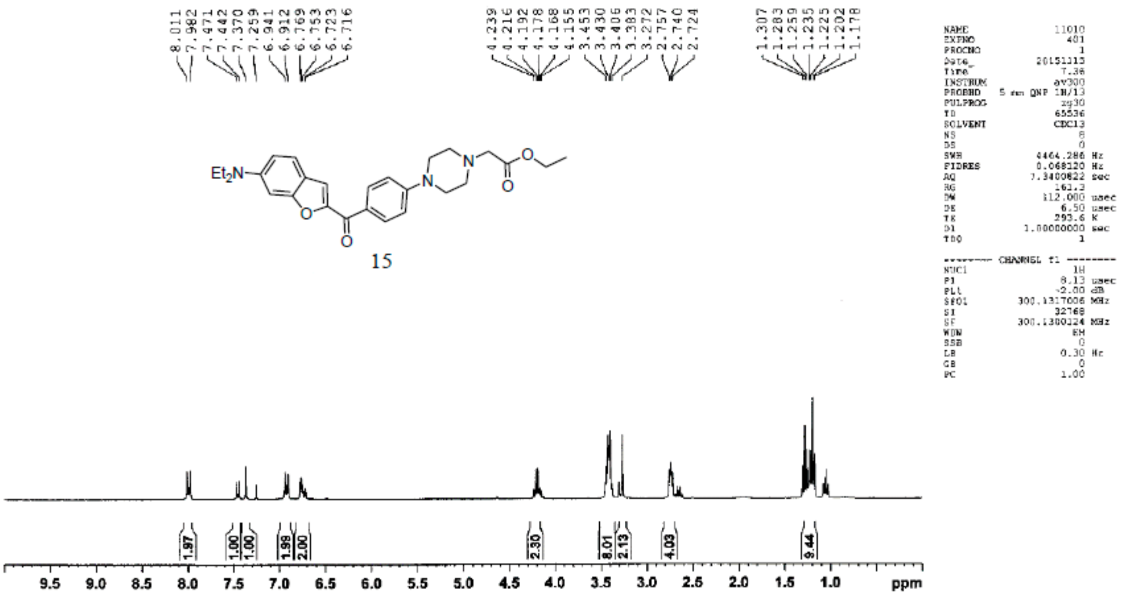

Figure S5. ^{1}H-NMR spectrum of compound 15.

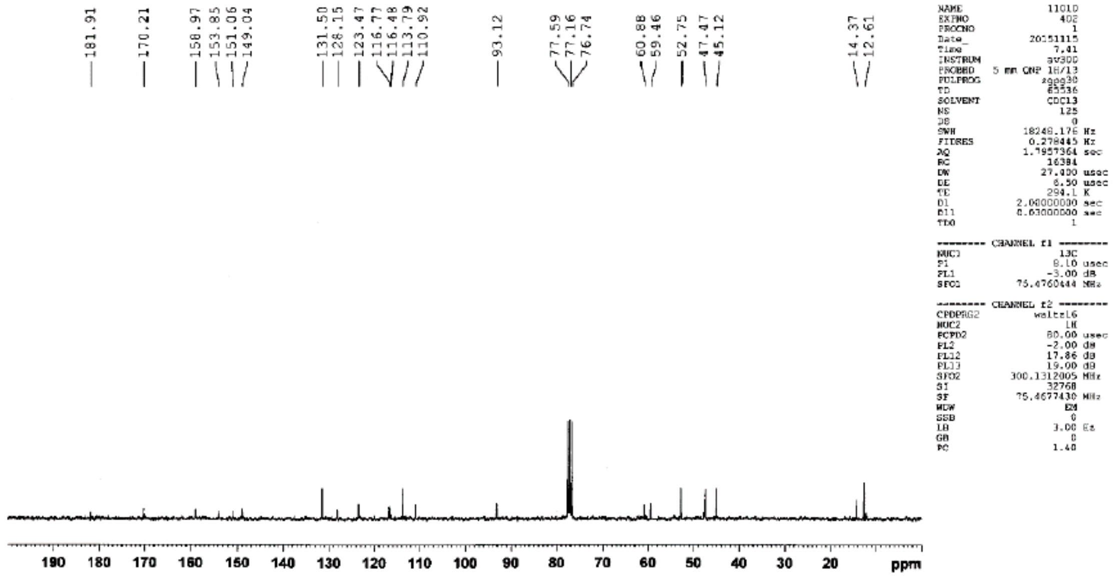

Figure S6. ^{13}C-NMR spectrum of compound 15.

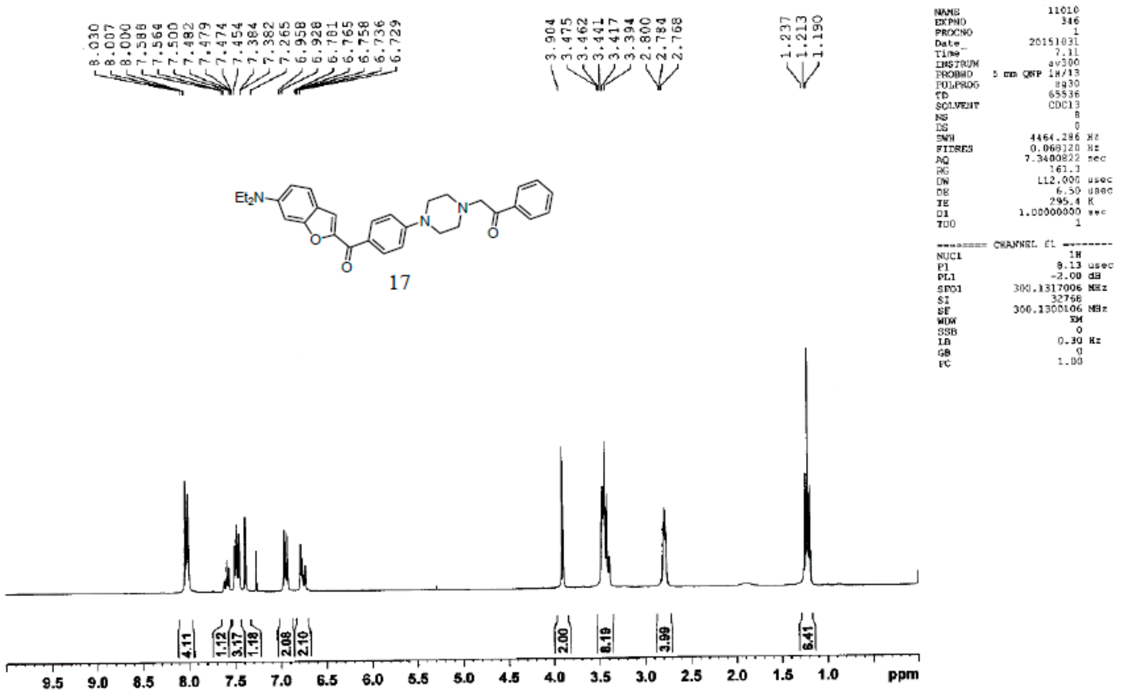

Figure S7. ^{1}H-NMR spectrum of compound 17.

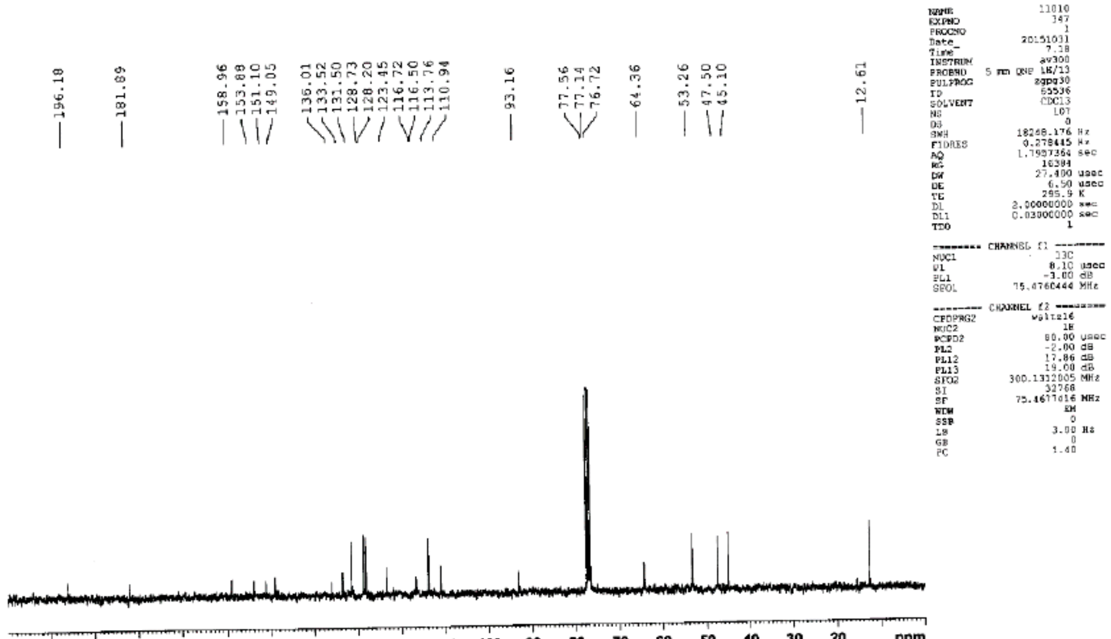

Figure S8. ^{13}C-NMR spectrum of compound 17.

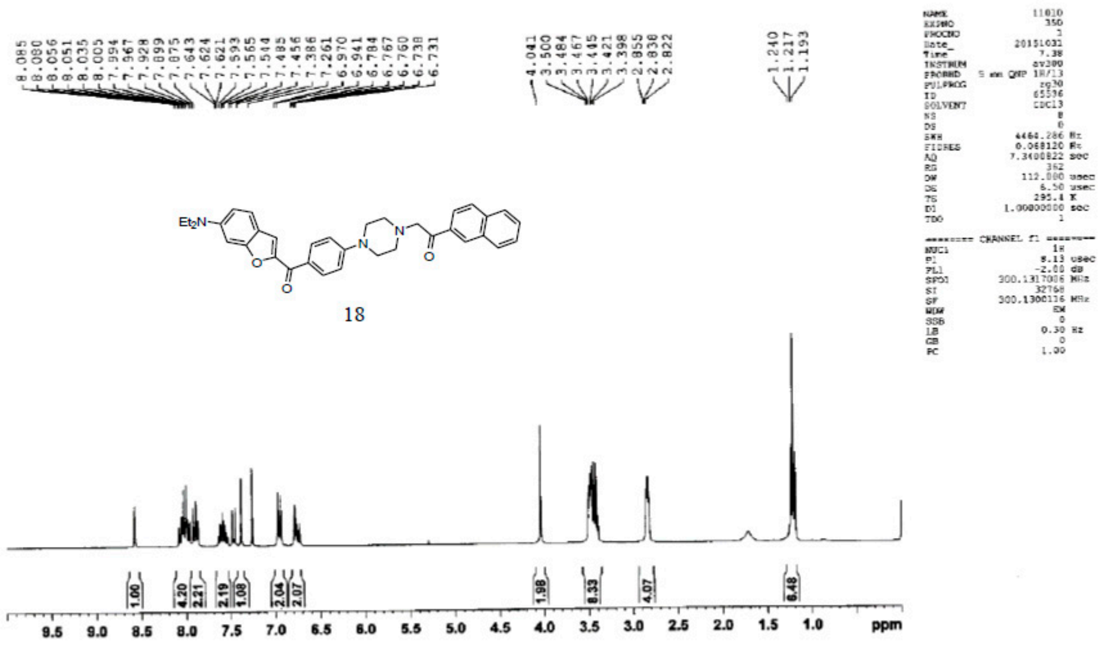

Figure S9. ^{1}H-NMR spectrum of compound 18.

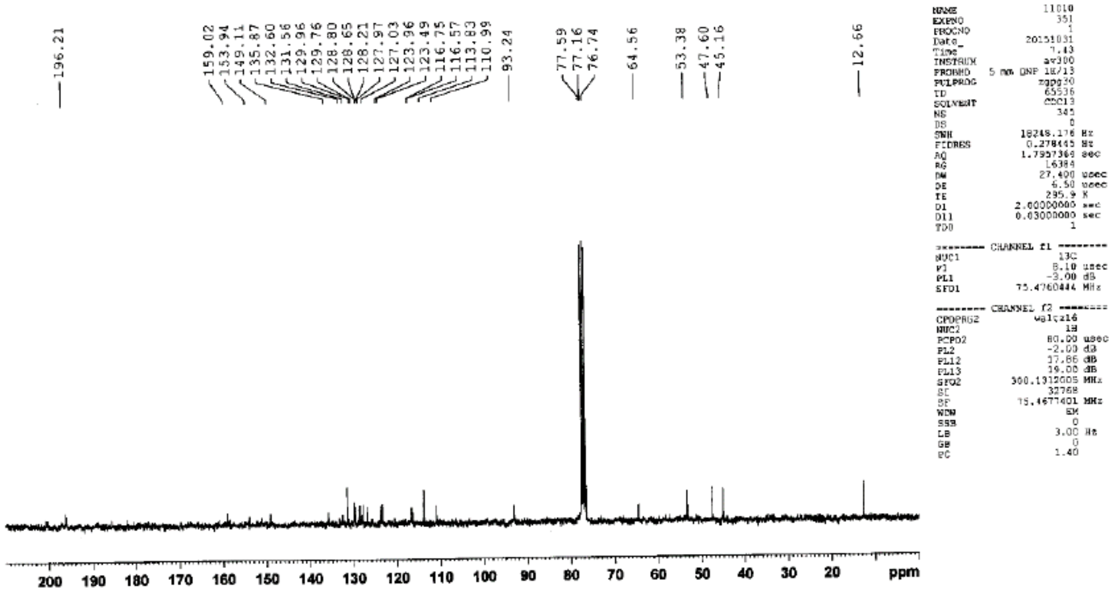

Figure S10. ^{13}C-NMR spectrum of compound 18.

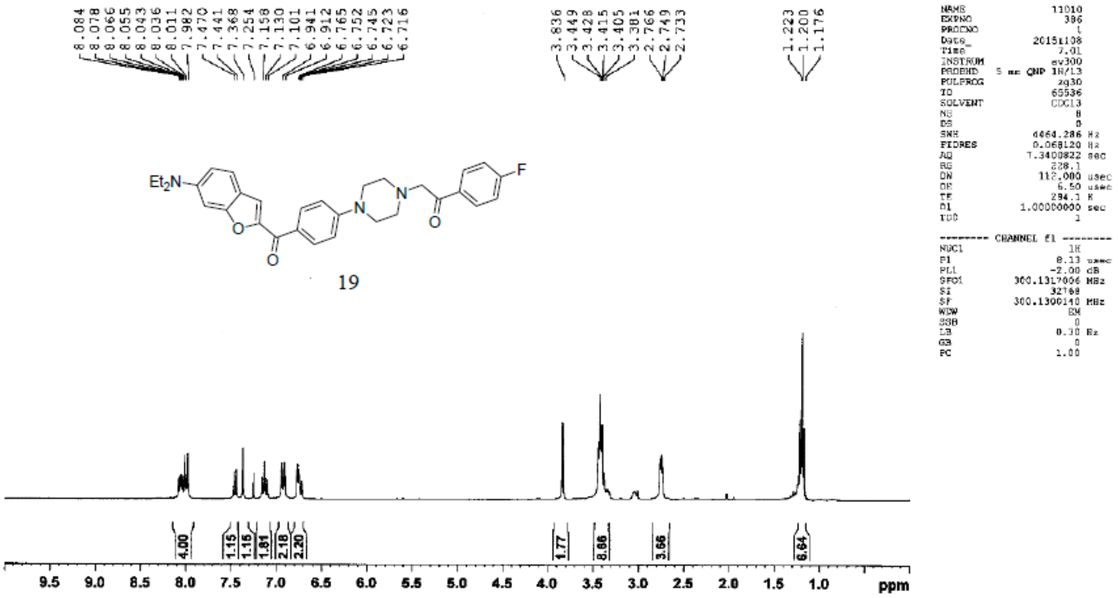

Figure S11. ^{1}H-NMR spectrum of compound 19.

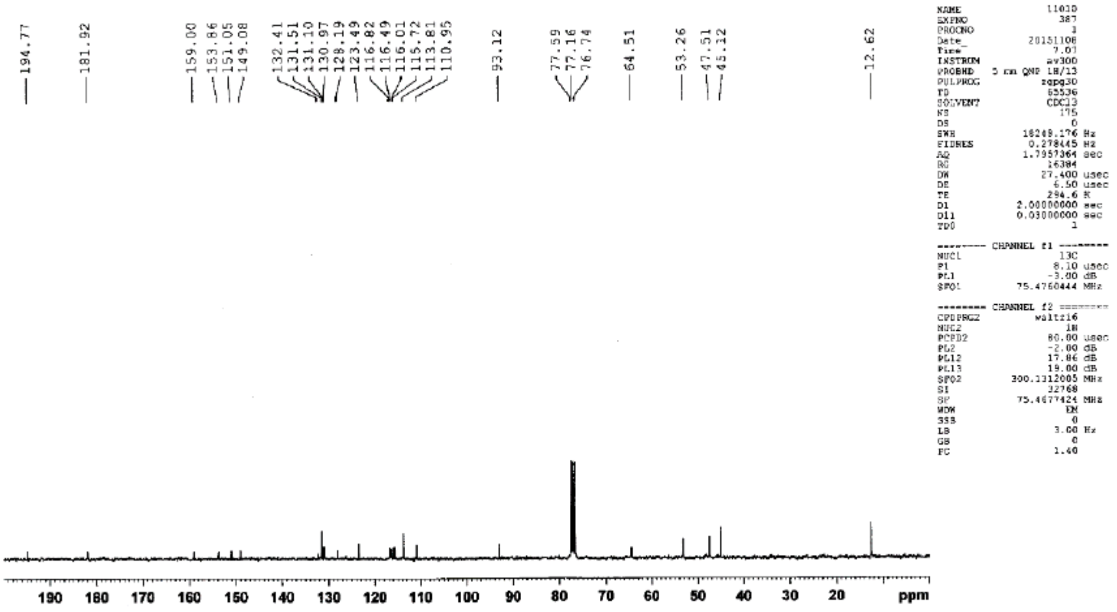

Figure S12. ^{13}C-NMR spectrum of compound 19.

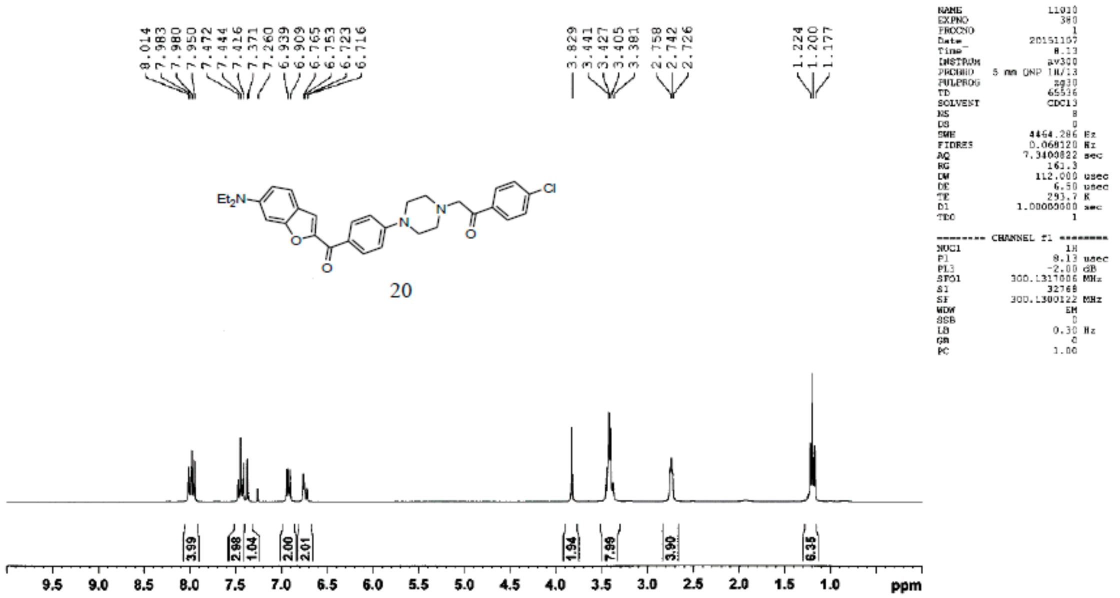

Figure S13. ^{1}H-NMR spectrum of compound 20.

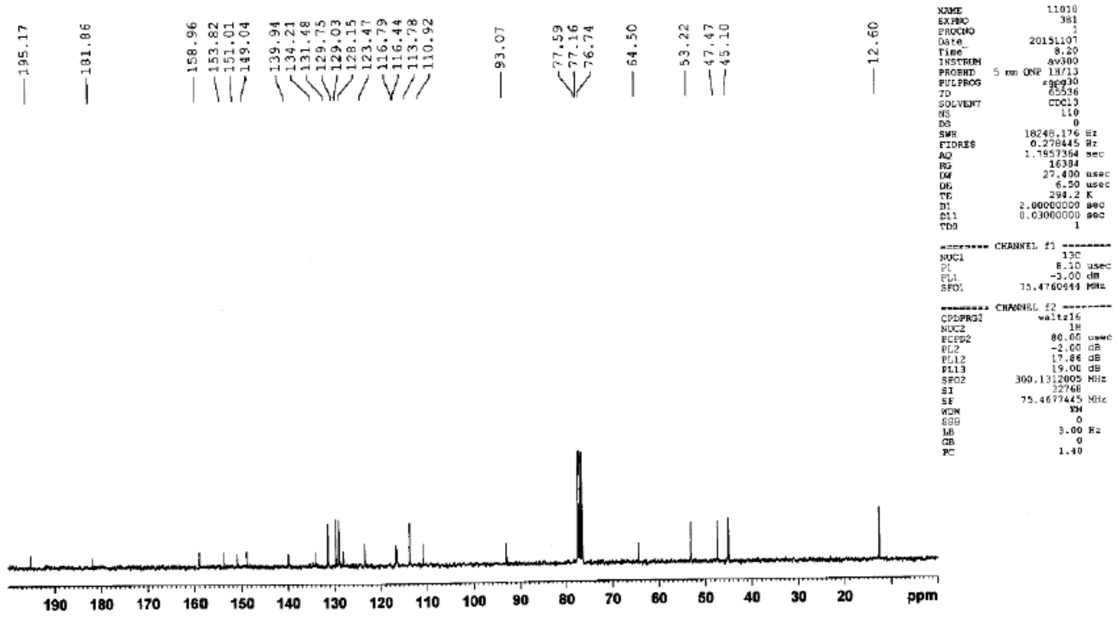

Figure S14. ^{13}C-NMR spectrum of compound 20.

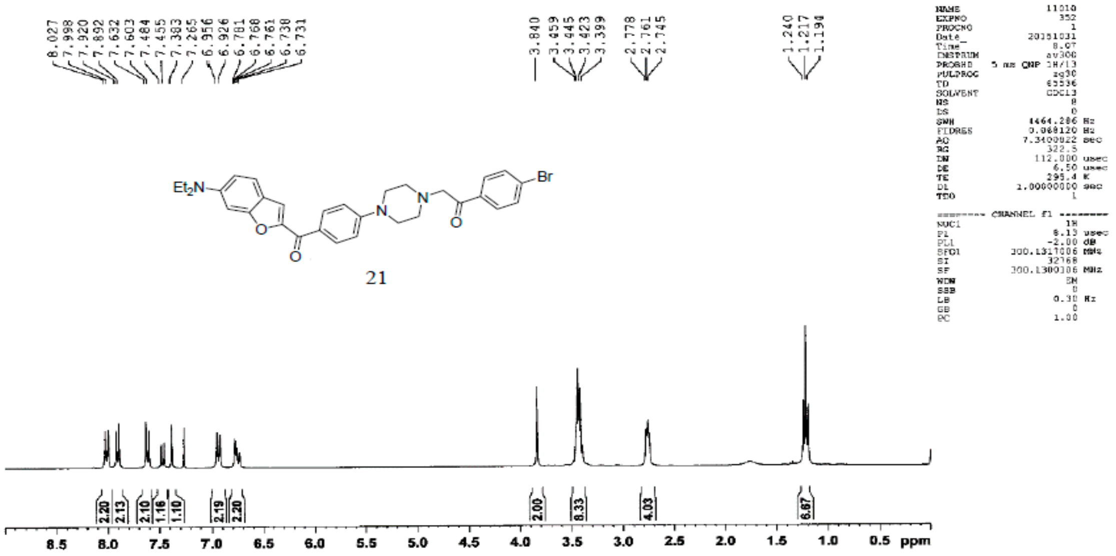

Figure S15. ^{1}H-NMR spectrum of compound 21.

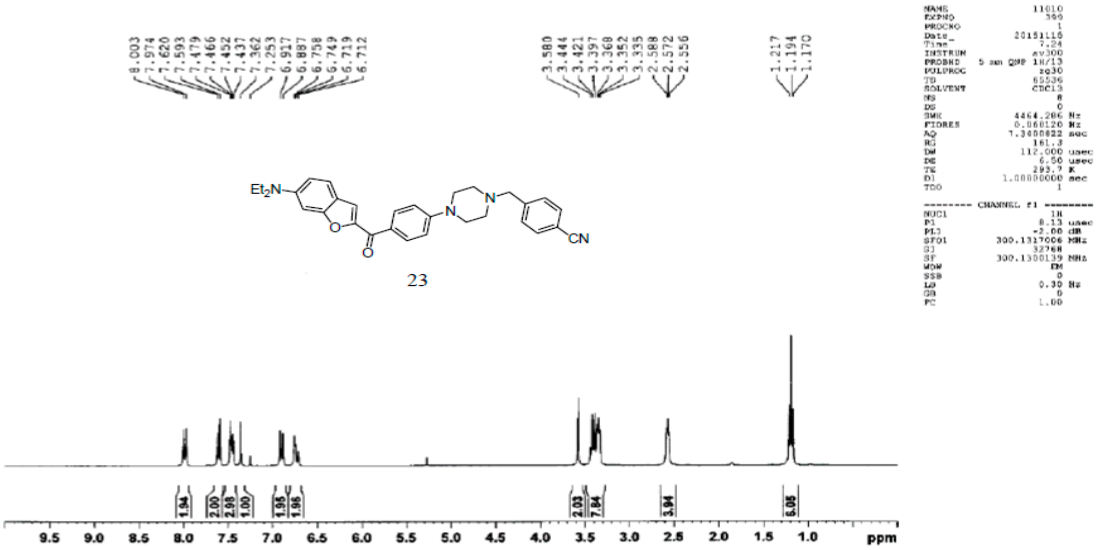

Figure S16. ^{1}H-NMR spectrum of compound 23.

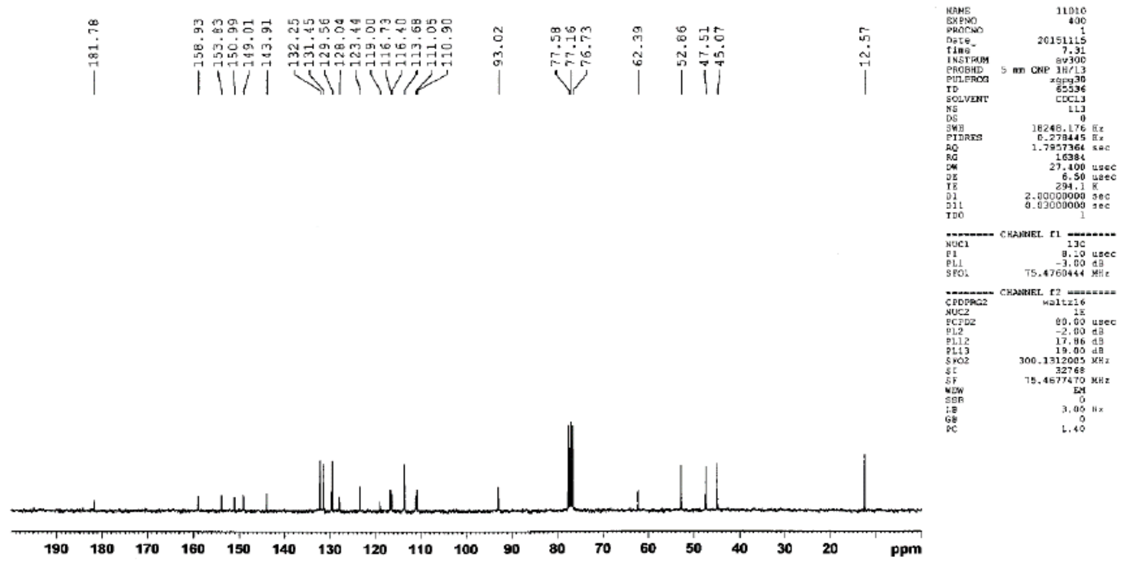

Figure S17. ^{13}C-NMR spectrum of compound 23.

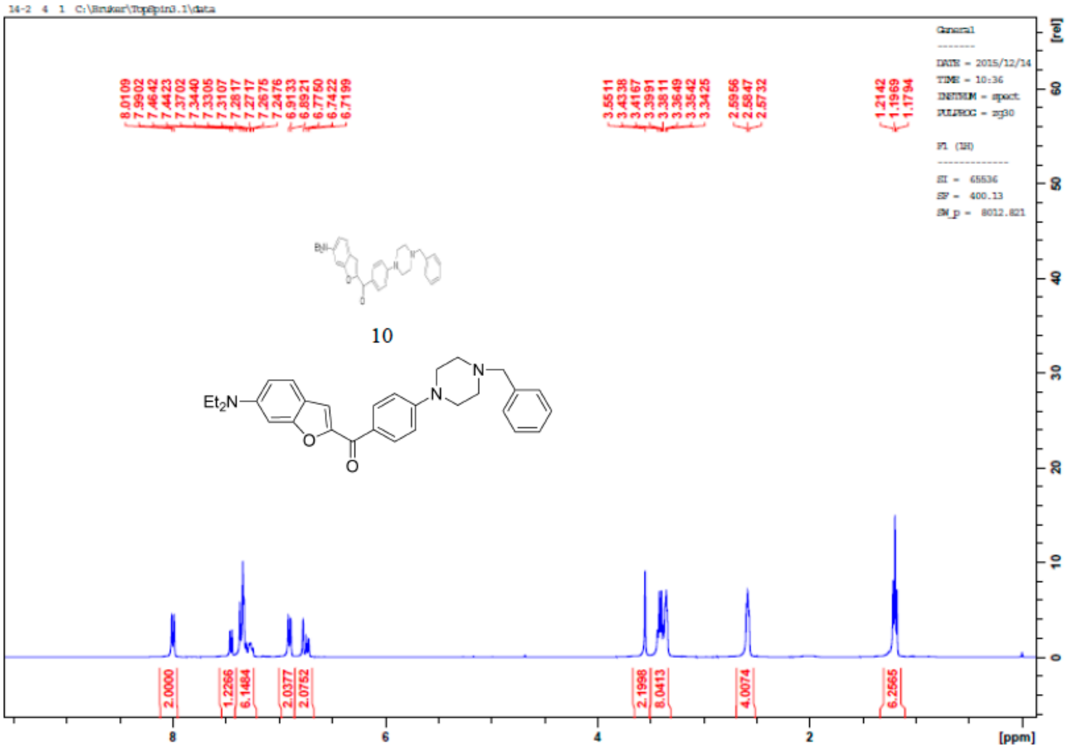

Figure S18. ^{1}H-NMR spectrum of compound 10.

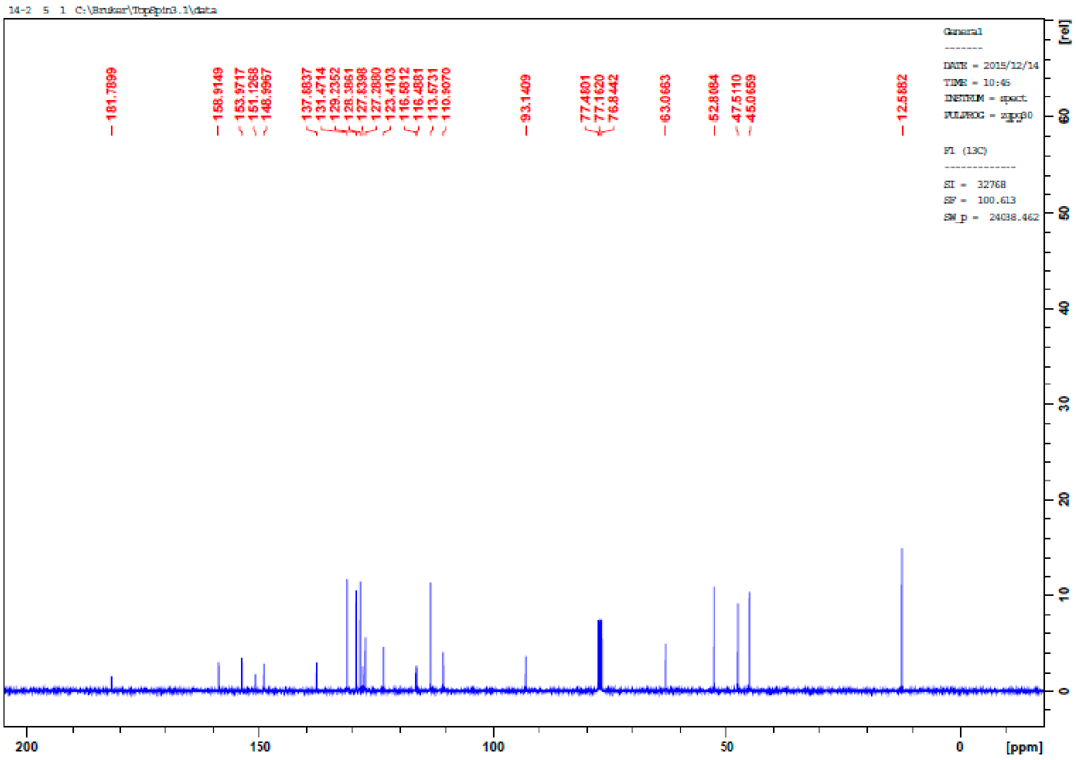

Figure S19. ^{13}C-NMR spectrum of compound 10.

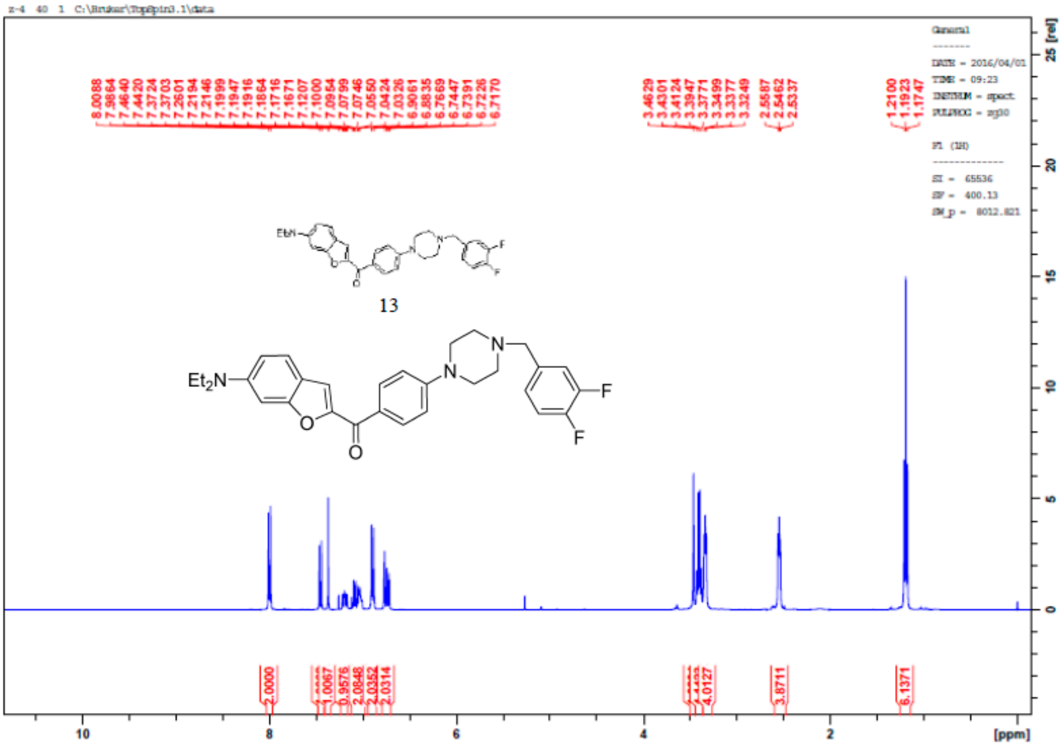

Figure S20. ^{1}H-NMR spectrum of compound 13.

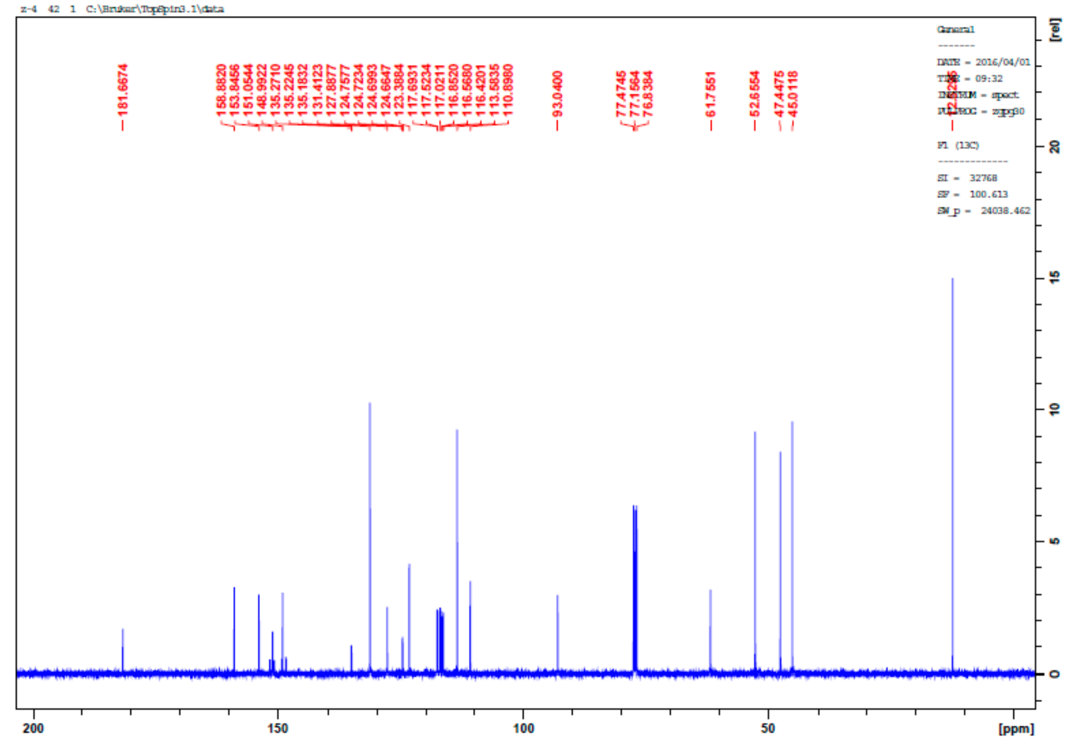

Figure S21. ^{13}C-NMR spectrum of compound 13.

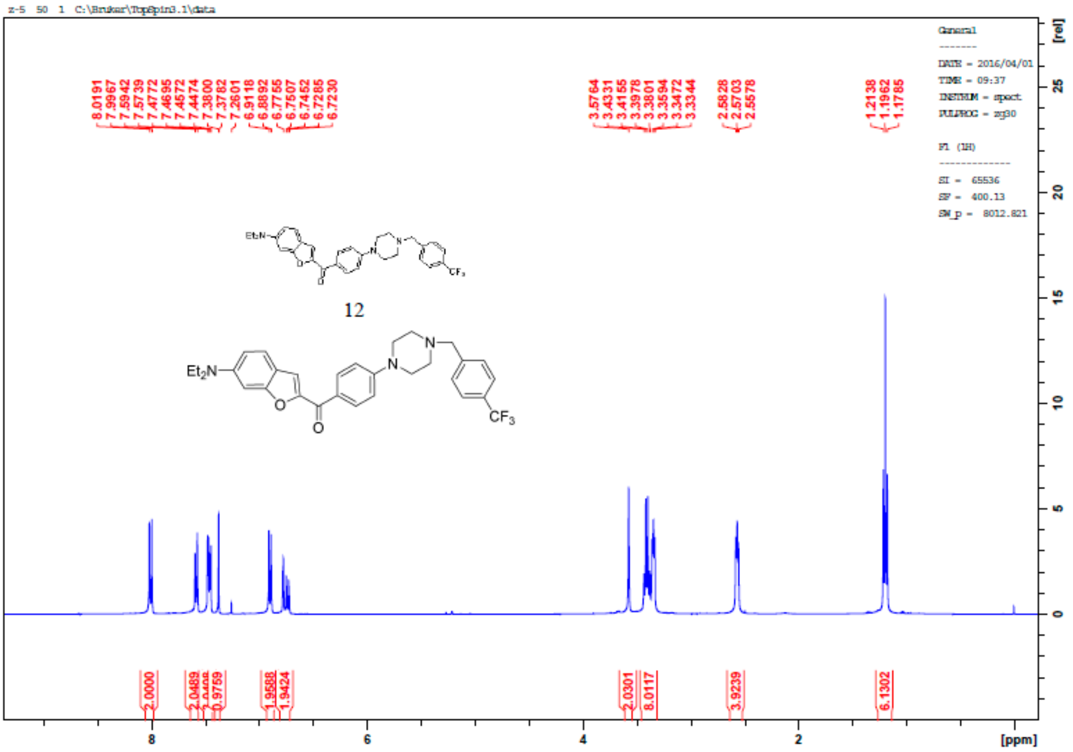

Figure S22. ^{1}H-NMR spectrum of compound 12.

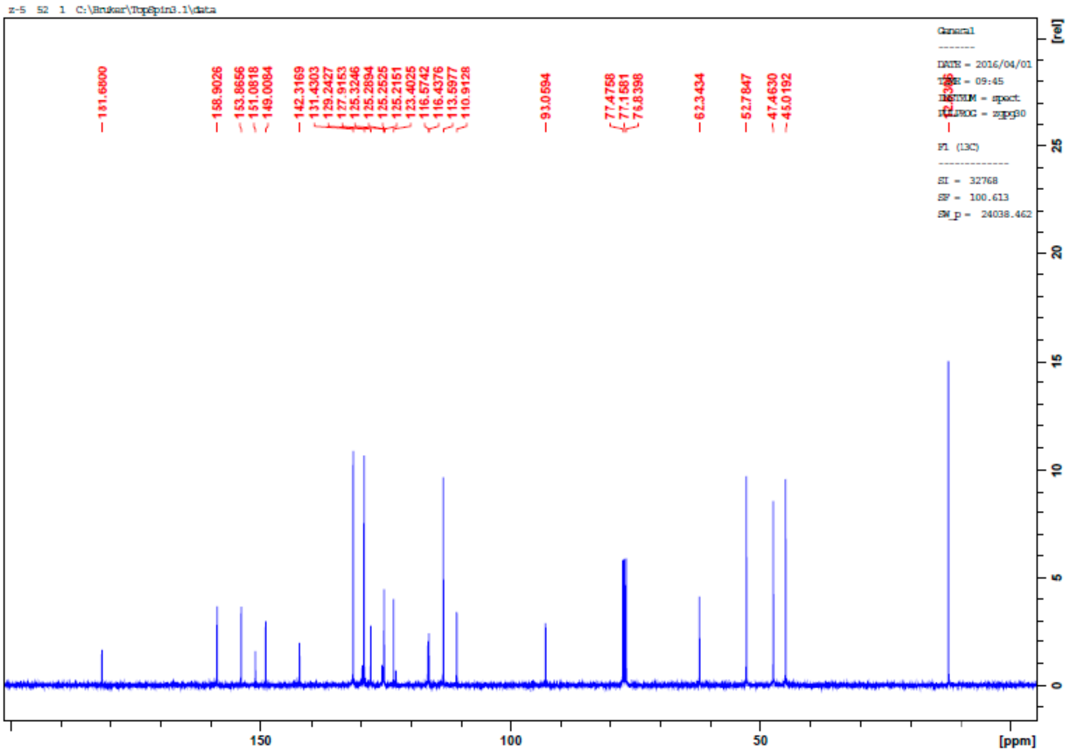

Figure S23. ^{13}C-NMR spectrum of compound 12.

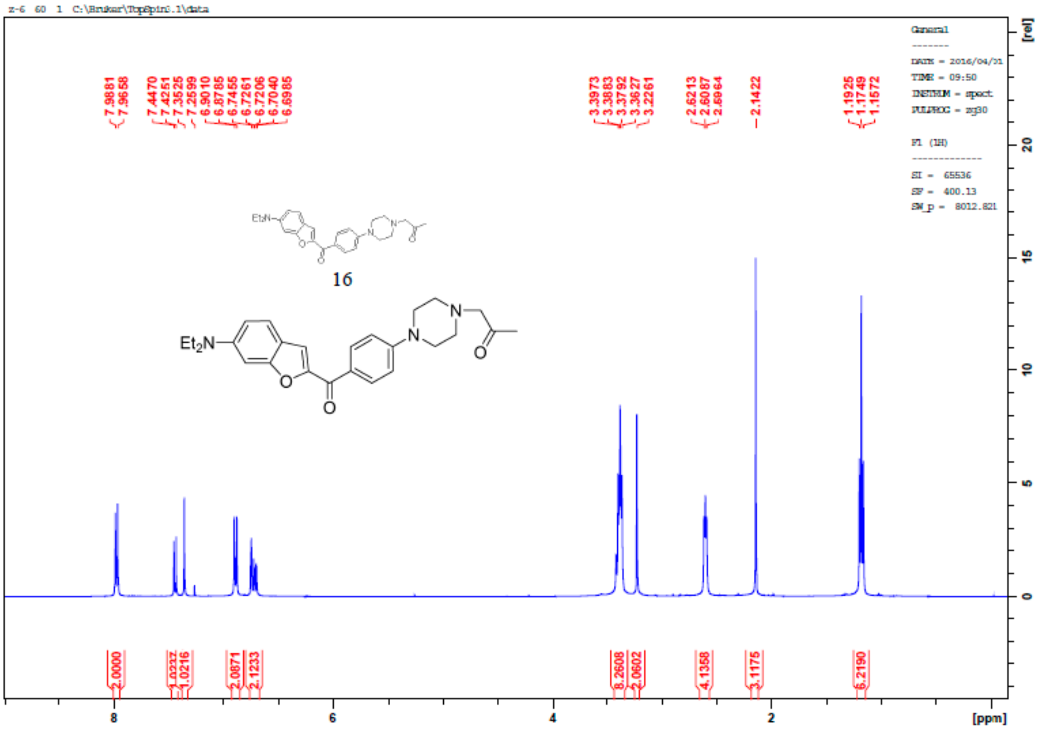

Figure S24. ^{1}H-NMR spectrum of compound 16.

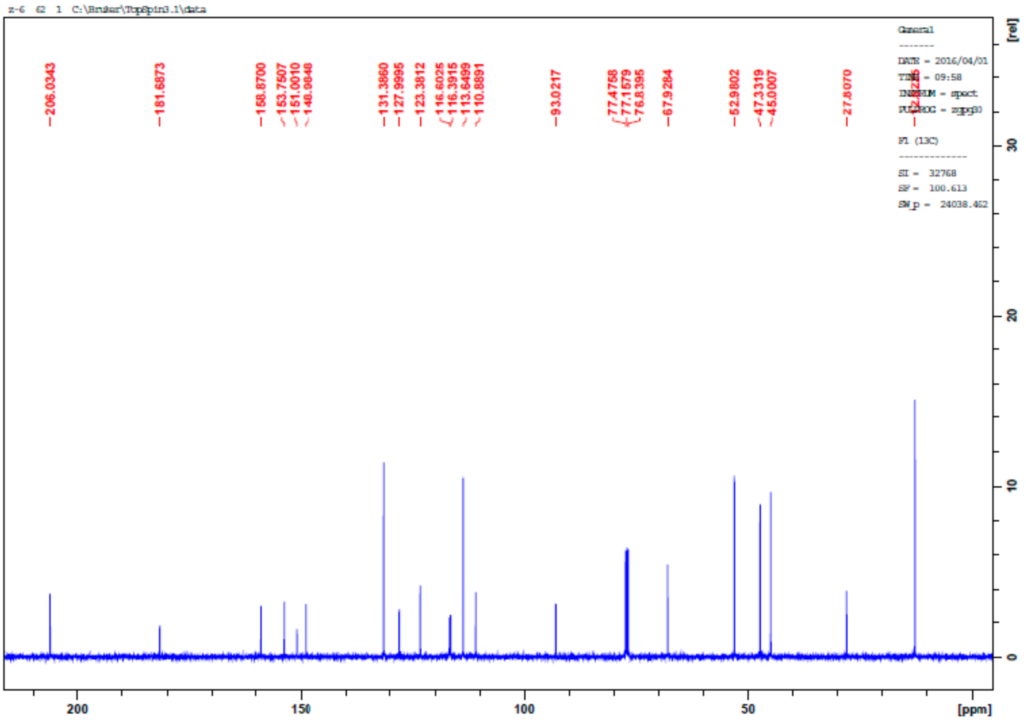

Figure S25. ^{13}C-NMR spectrum of compound 16.

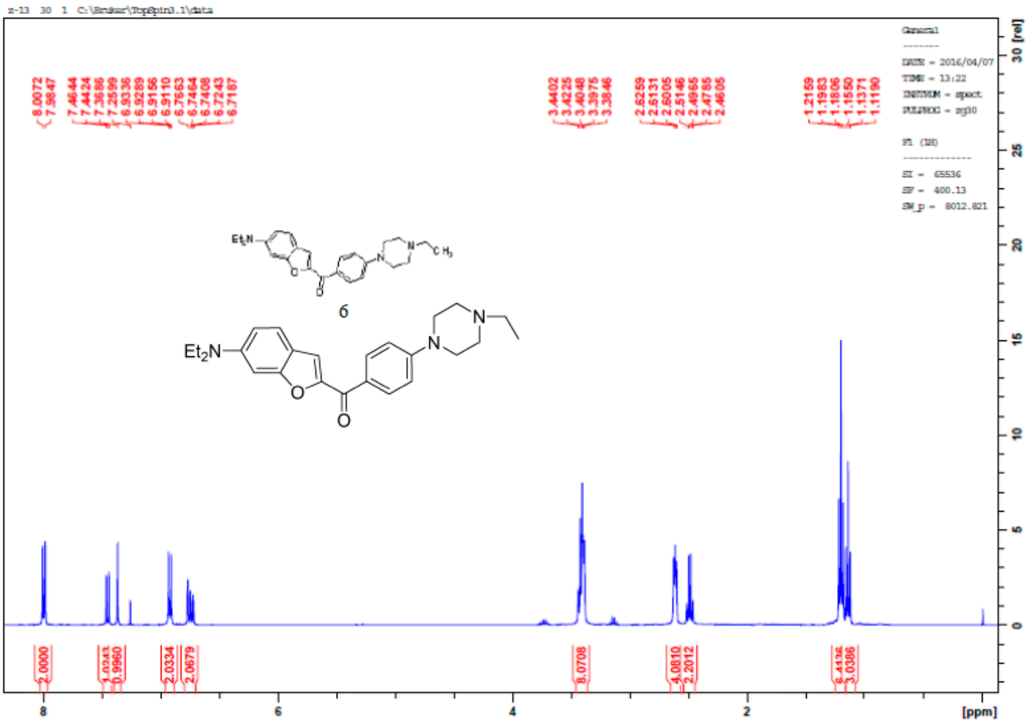

Figure S26. ^{1}H-NMR spectrum of compound 6.

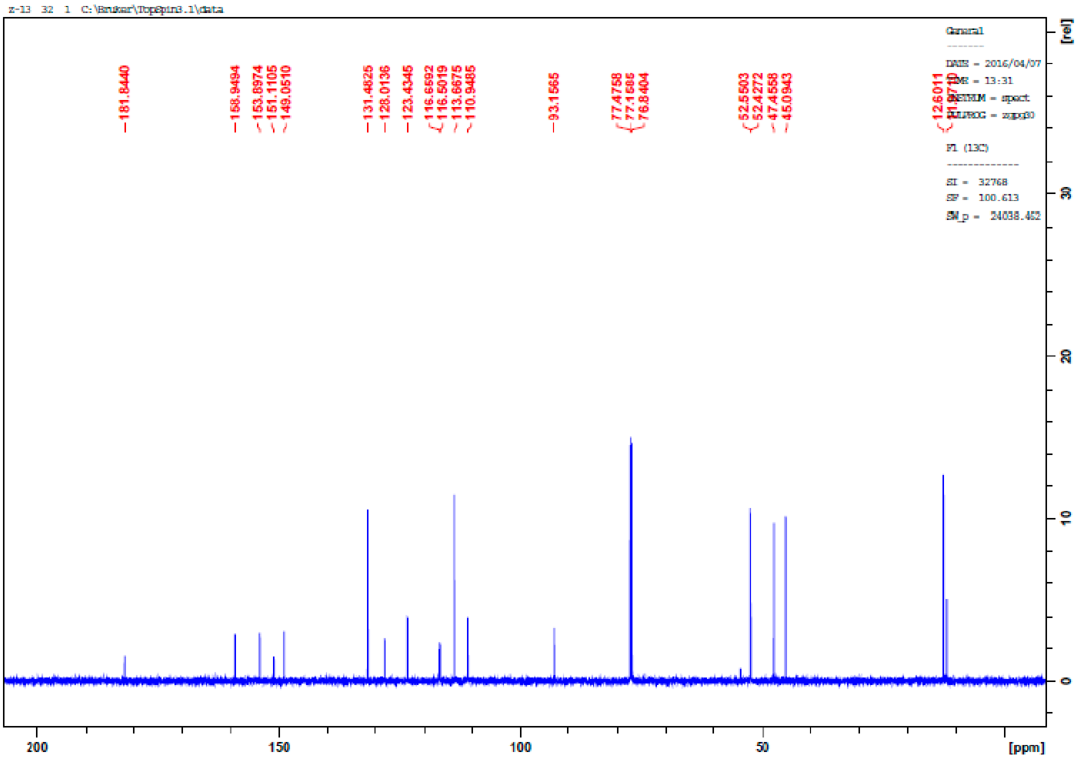

Figure S27. ^{13}C-NMR spectrum of compound 6.

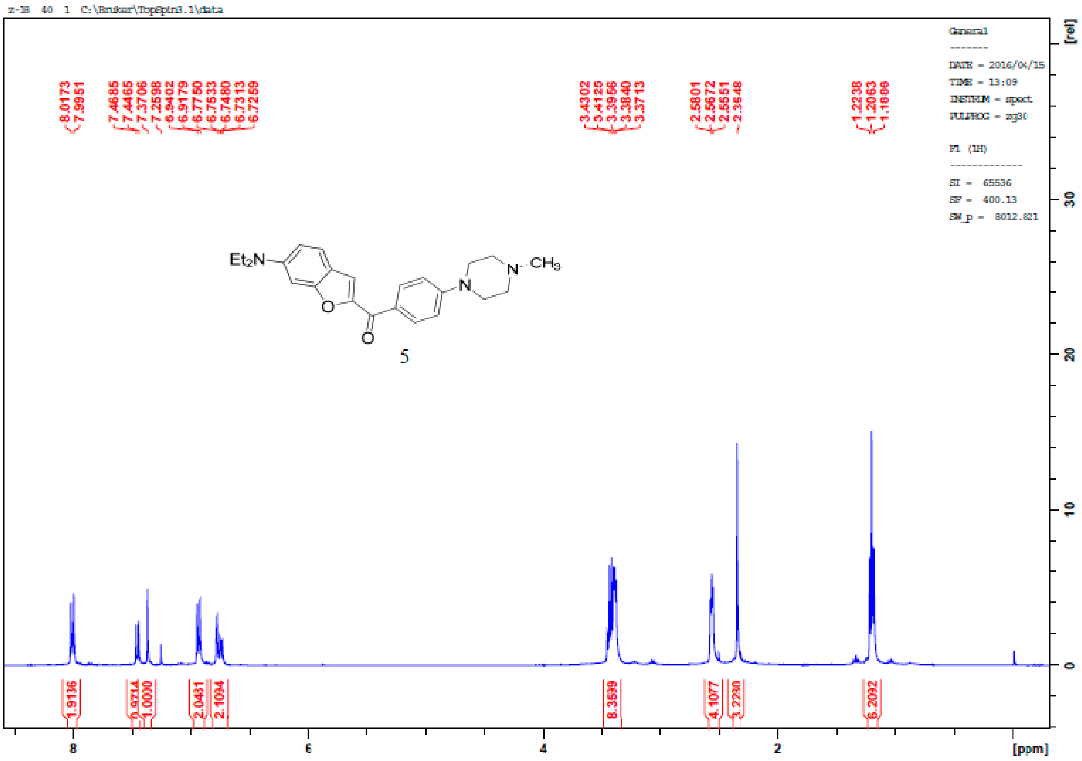

Figure S28. ^{1}H-NMR spectrum of compound 5

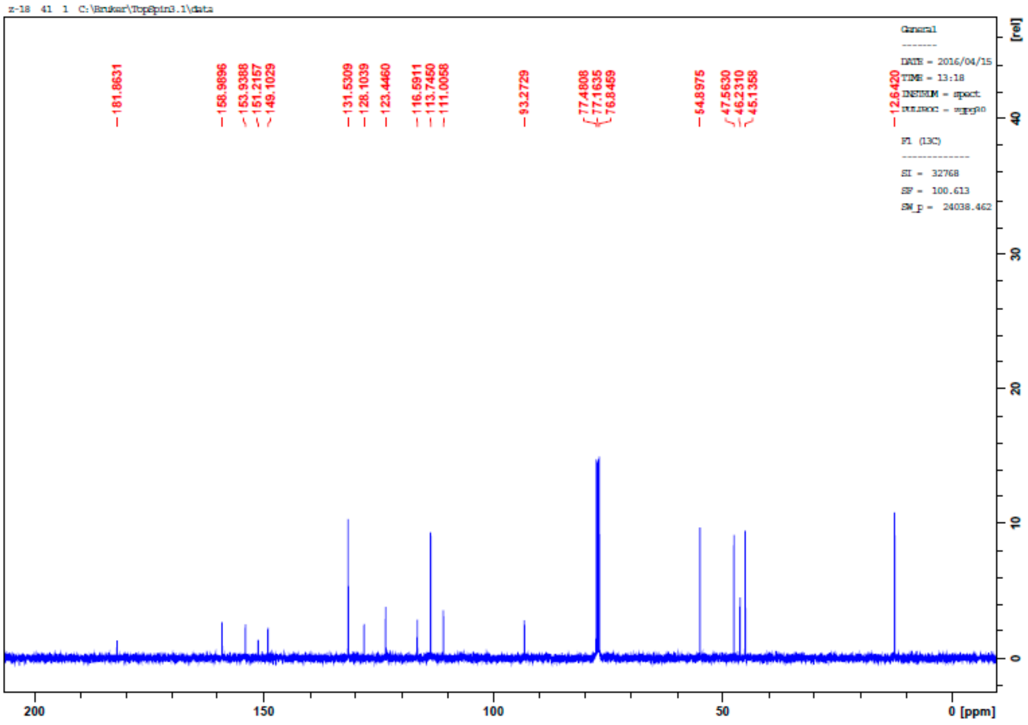

Figure S29. ^{13}C-NMR spectrum of compound 5.

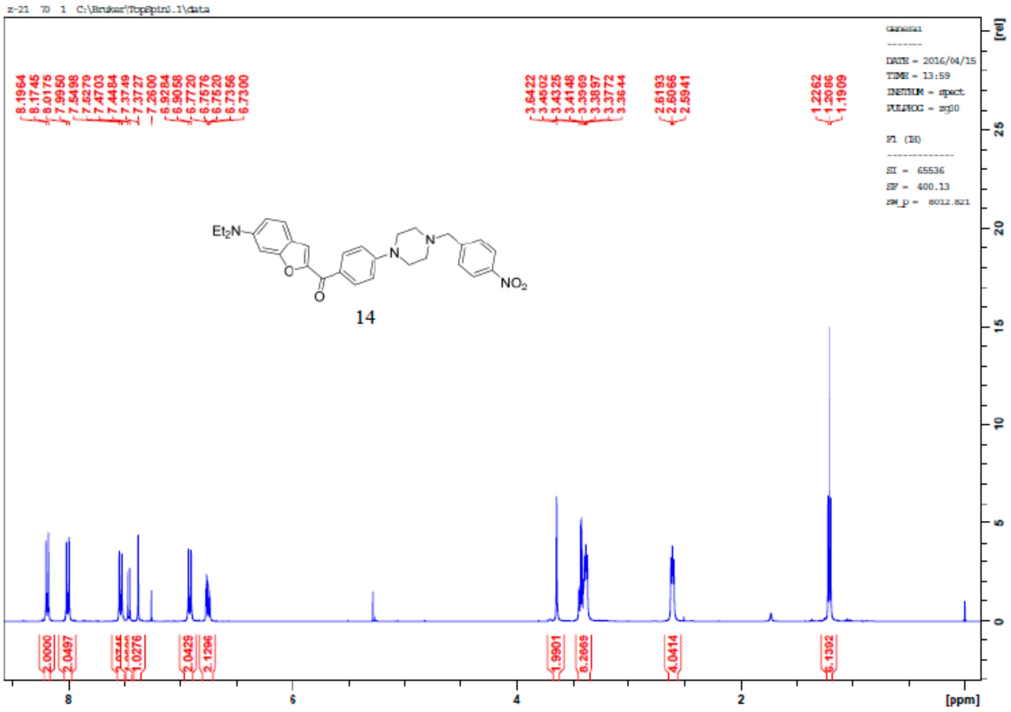

Figure S30. ^{1}H-NMR spectrum of compound 14.

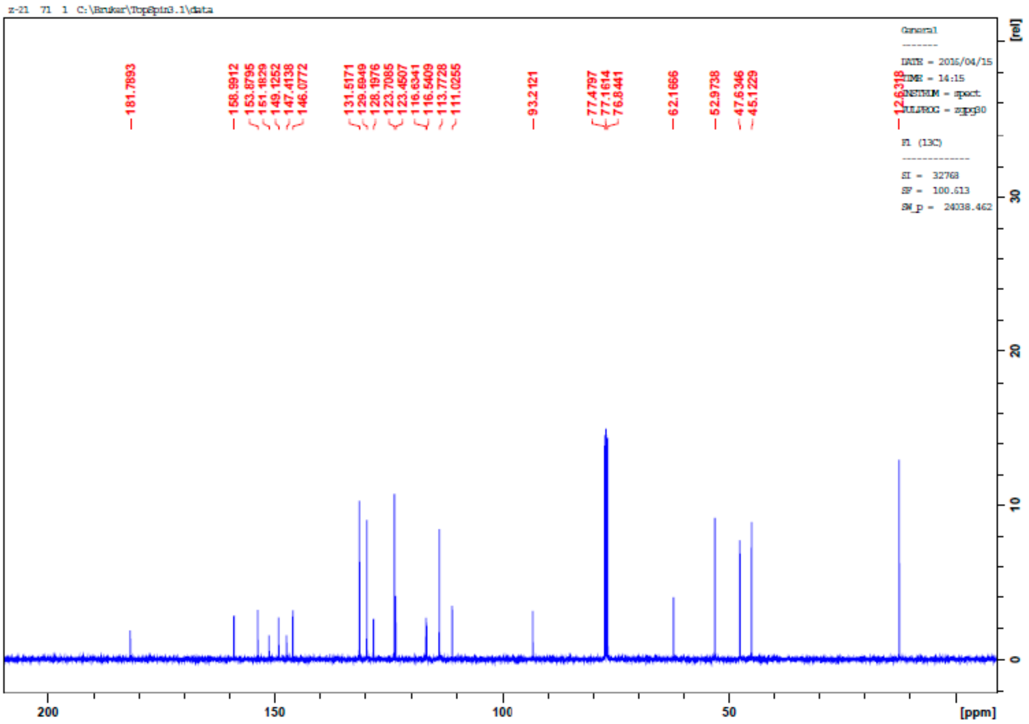

Figure S31. ^{13}C-NMR spectrum of compound 14.

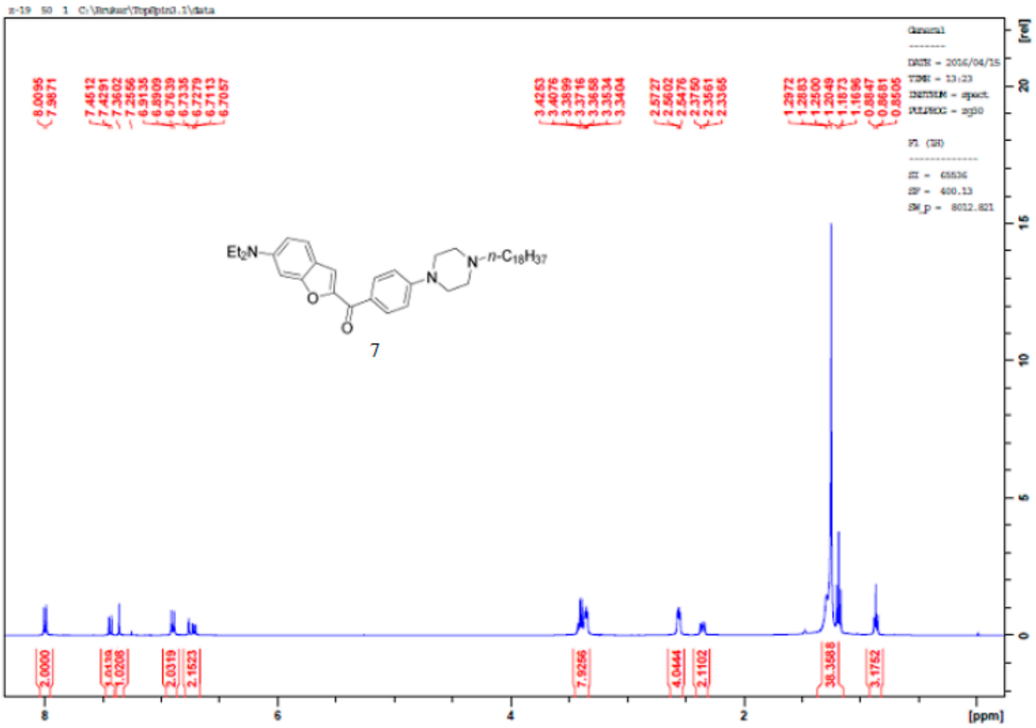

Figure S32. ^{1}H-NMR spectrum of compound 7.

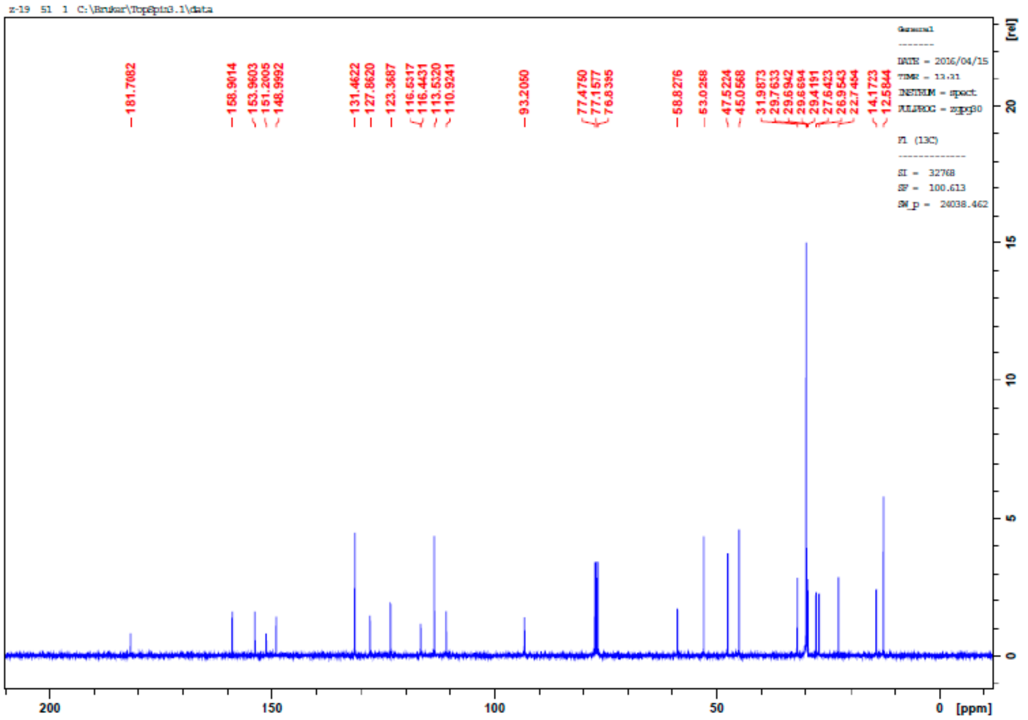

Figure S33. ^{13}C-NMR spectrum of compound 7.

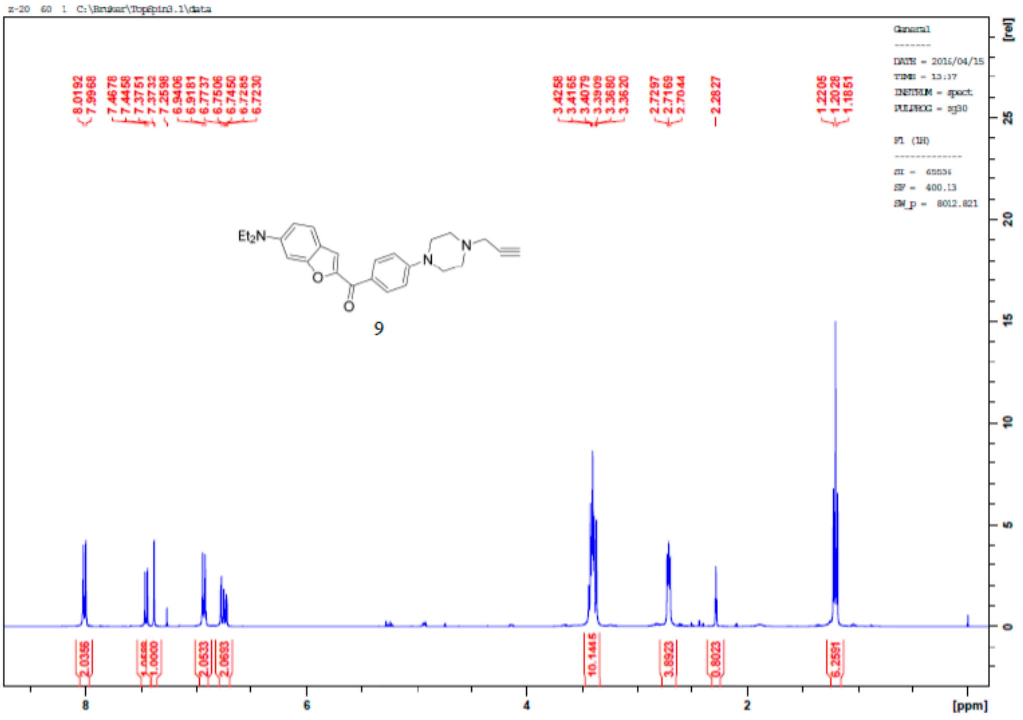

Figure S34. ^{1}H-NMR spectrum of compound 9.

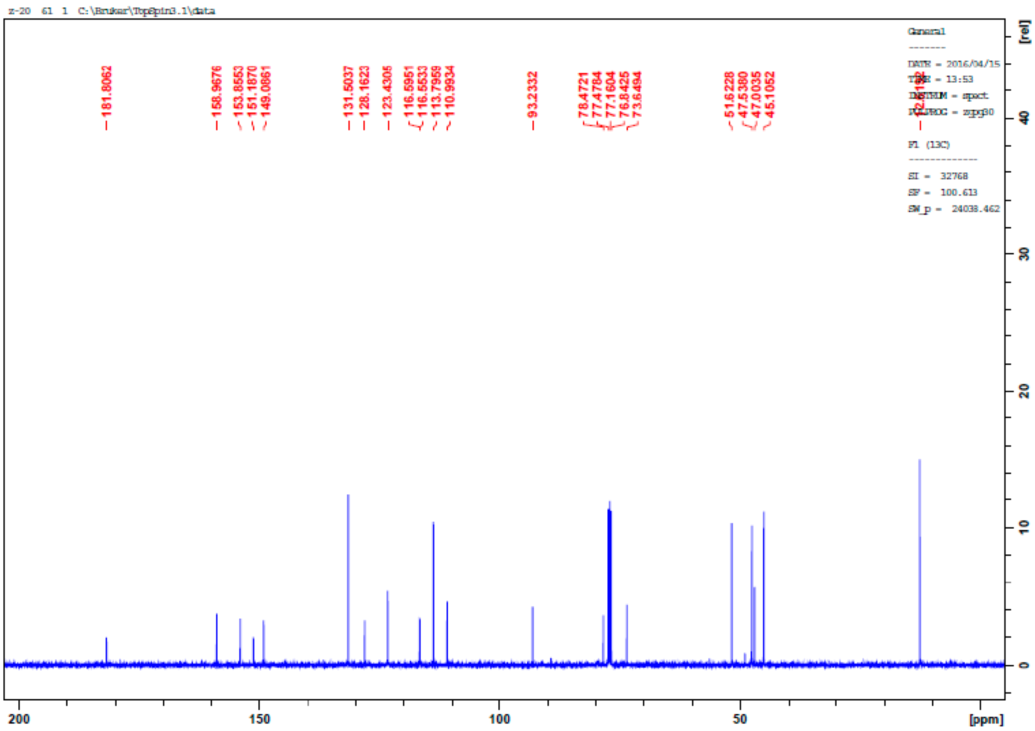

Figure S35. ^{13}C-NMR spectrum of compound 9.

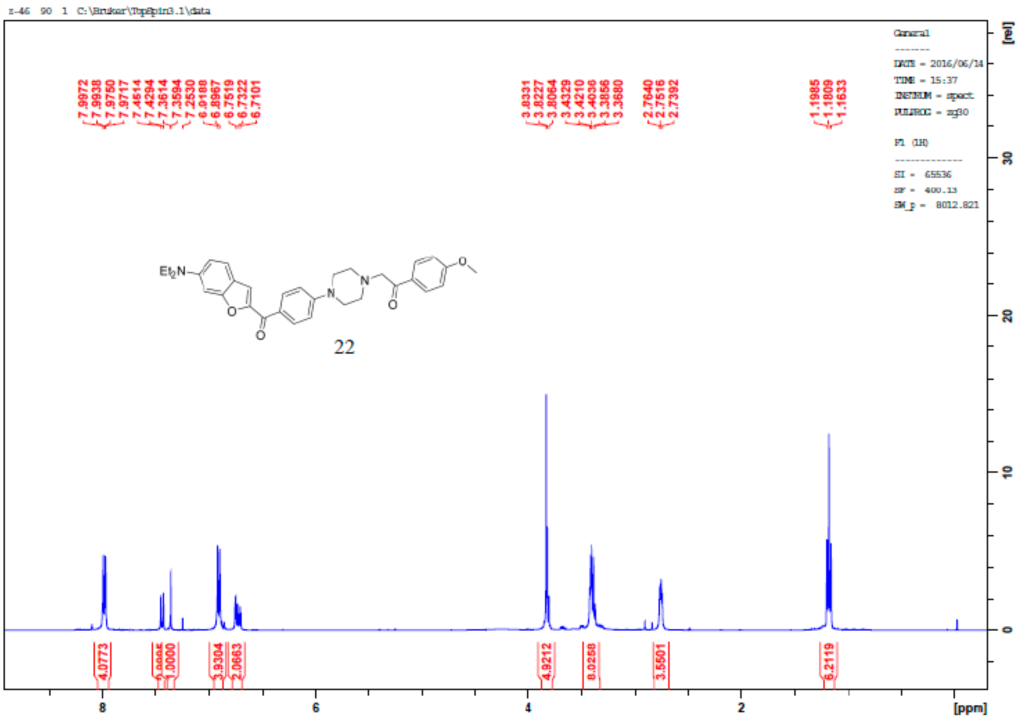

Figure S36. ^{1}H-NMR spectrum of compound 22.

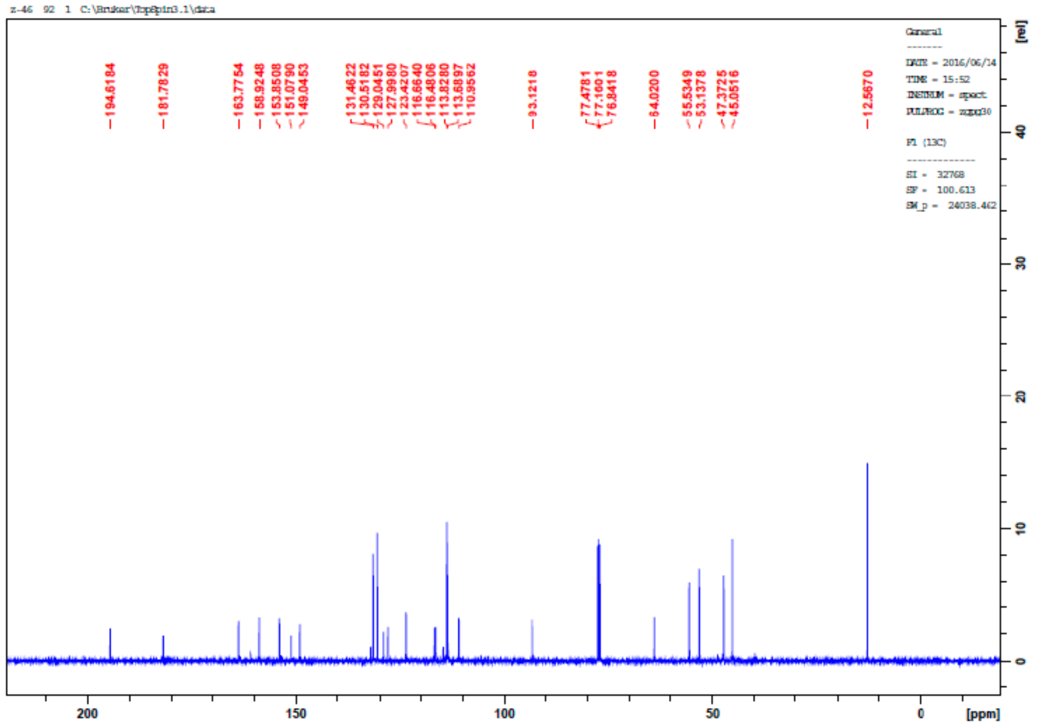

Figure S37. ^{13}C-NMR spectrum of compound 22.

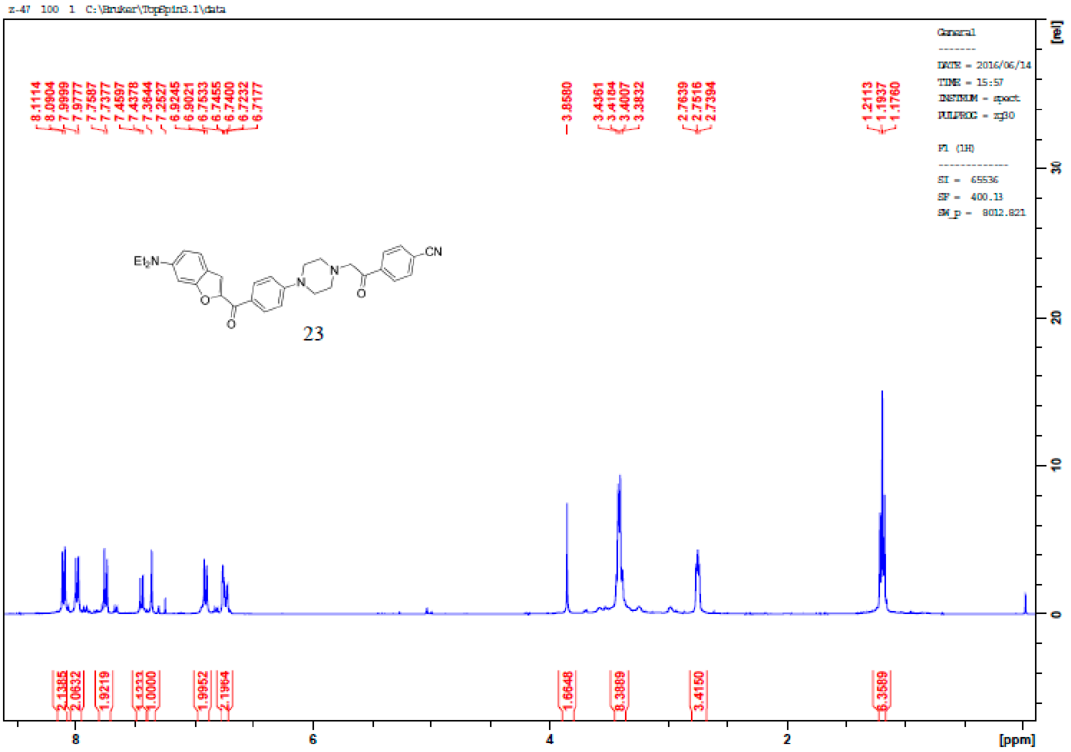

Figure S38. ^{1}H-NMR spectrum of compound 23.

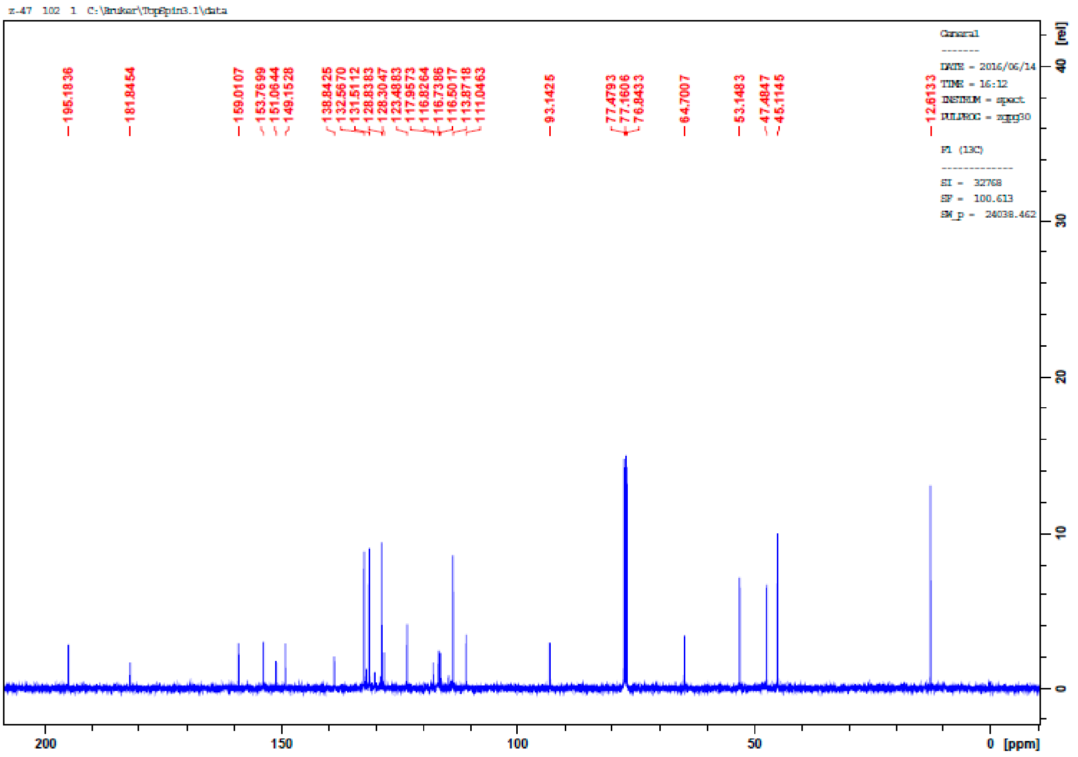

Figure S39. ^{13}C-NMR spectrum of compound 23.

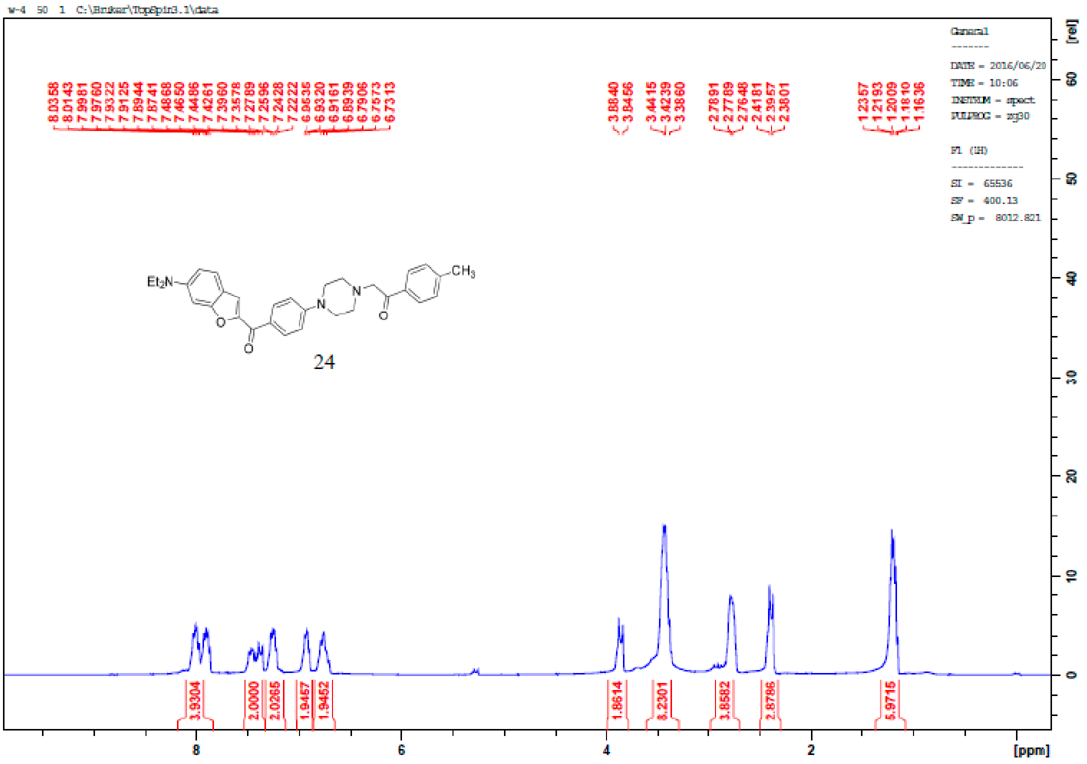

Figure S40. ^{1}H-NMR spectrum of compound 24.

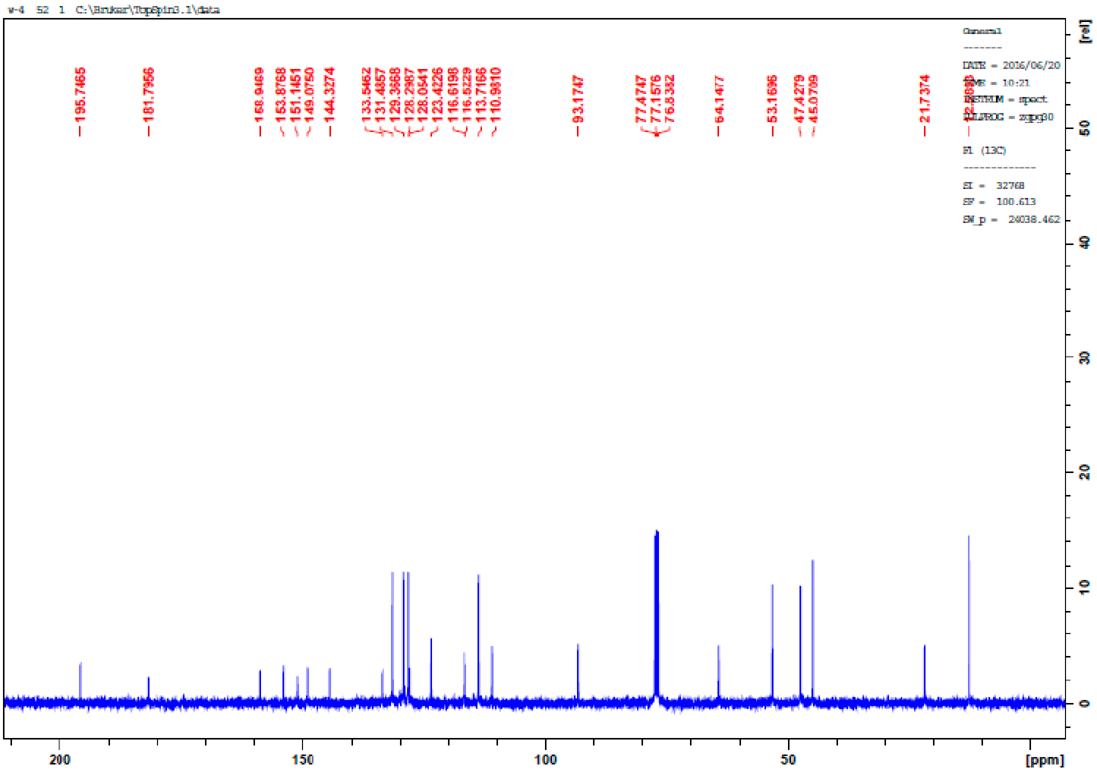

Figure S41. ^{13}C-NMR spectrum of compound 24.

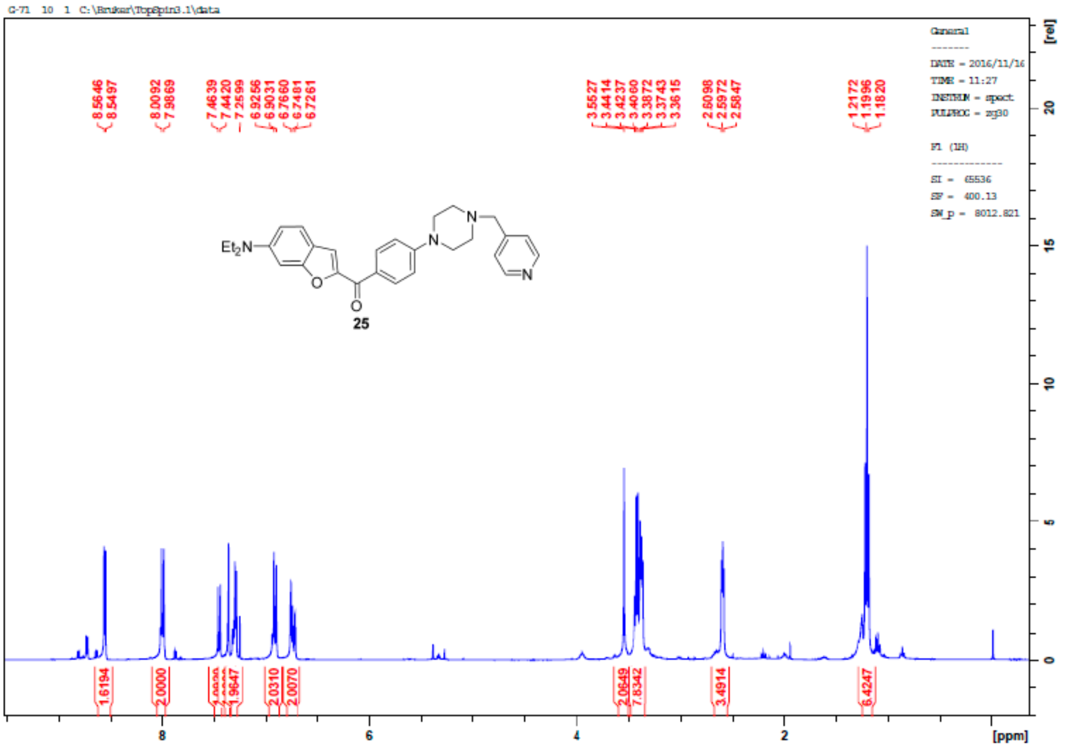

Figure S42. ^{1}H-NMR spectrum of compound 25.

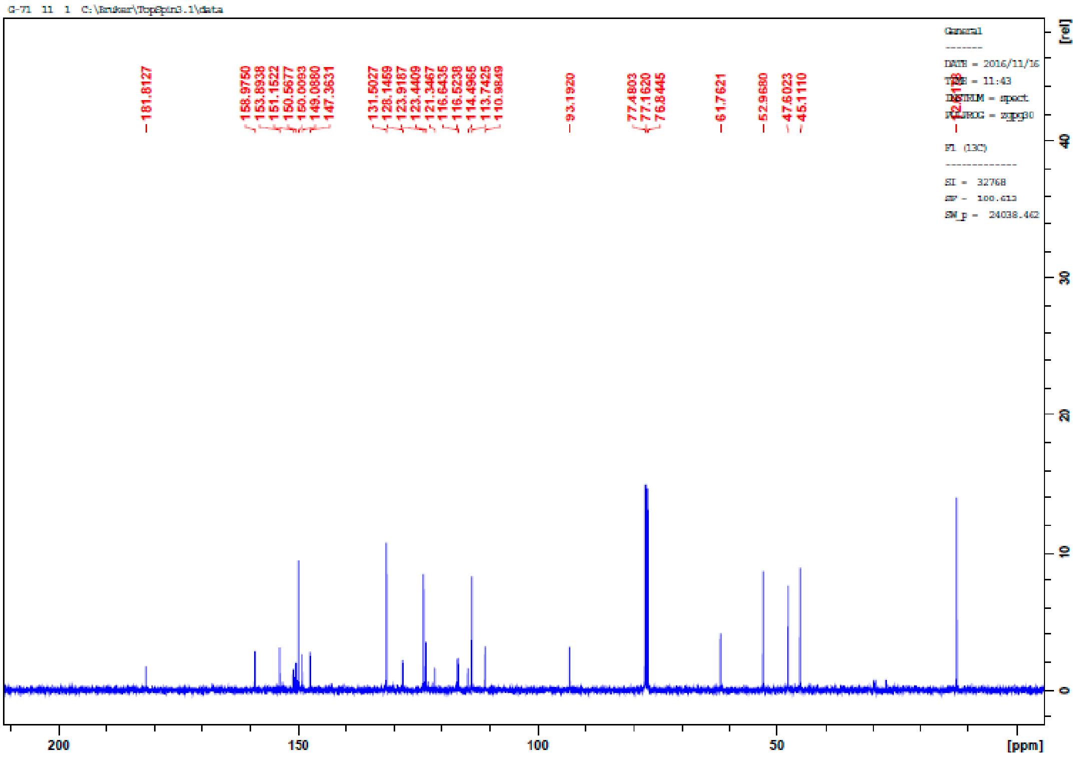

Figure S43. ^{13}C-NMR spectrum of compound 25.

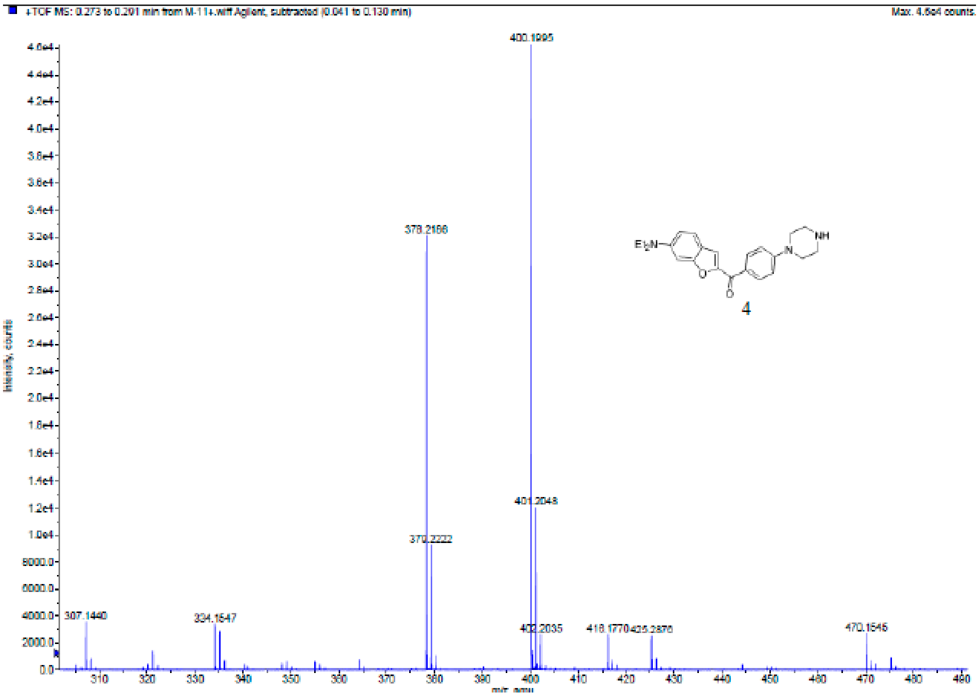

Elemental composition calculator

```
Target m/z:          +400.1995      amu
Tolerance:           +10.0000       ppm
Result type:       Elemental
Max num of results:        100
Min DBE:     -0.5000       Max DBE:          +50.0000
Electron state:    OddAndEven
Num of charges:    1
Add water:         N/A
Add proton:        N/A
File Name:         N-11+.wiff
```

	Elements	Min Number	Max Number
1	C	0	23
2	F	0	0
3	H	0	27
4	K	0	0
5	N	0	3
6	Na	0	1
7	O	0	2
8	Pt	0	0
9	S	0	0

	Formula	Calculated m/z (amu)	mDa Error	PPM Error	DBE
1	C23 H27 N3 O2 Na	400.1995	-0.0485	-0.1211	11.5

Figure S44. HRMS spectrum of compound 4.

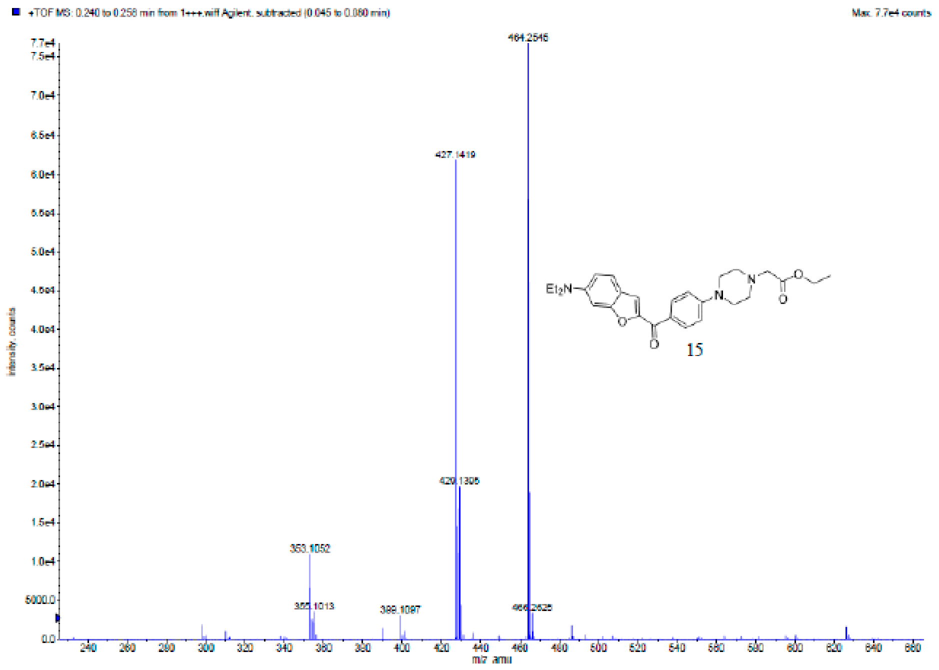

Elemental composition calculator

```
Target m/z:          +464.2545      amu
Tolerance:           +100.0000      ppm
Result type:       Elemental
Max num of results:        100
Min DBE:     -0.5000       Max DBE:          +50.0000
Electron state:    Even
Num of charges:    1
Add water:         N/A
Add proton:        N/A
File Name:         1+++.wiff
```

	Elements	Min Number	Max Number
1	Br	0	0
2	C	0	27
3	F	0	0
4	H	0	34
5	Ir	0	0
6	N	0	3
7	Na	0	1
8	O	0	4
9	S	0	0

	Formula	Calculated m/z (amu)	mDa Error	PPM Error	DBE
1	C27 H34 N3 O4	464.2543	0.1166	0.2512	12.5

Figure S45. HRMS spectrum of compound 15.

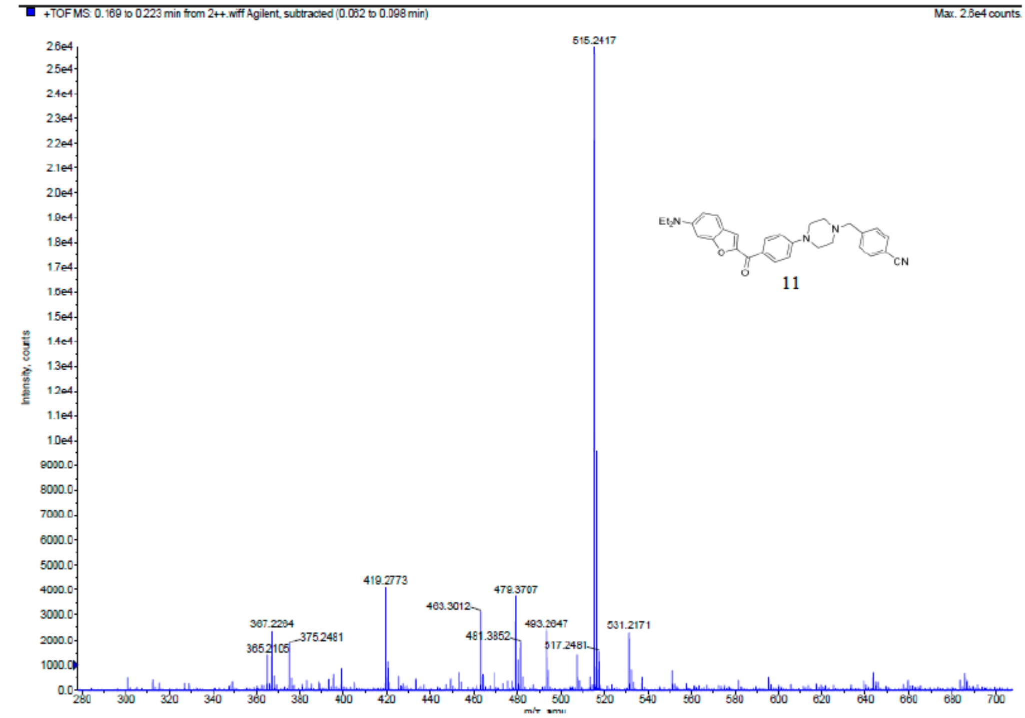

Elemental composition calculator

```
Target m/z:        +515.2417      amu
Tolerance:         +100.0000      ppm
Result type:      Elemental
Max num of results:       100
Min DBE:     -0.5000      Max DBE:          +50.0000
Electron state:   Even
Num of charges:   1
Add water:        N/A
Add proton:       N/A
File Name:        2++.wiff
```

	Elements	Min Number	Max Number
1	Br	0	0
2	C	0	31
3	F	0	0
4	H	0	32
5	Ir	0	0
6	N	0	4
7	Na	0	1
8	O	0	2
9	S	0	0

	Formula	Calculated m/z (amu)	mDa Error	PPM Error	DBE
1	C31 H32 N4 O2 Na	515.2417	-0.0477	-0.0926	17.5

Figure S46. HRMS spectrum of compound 11.

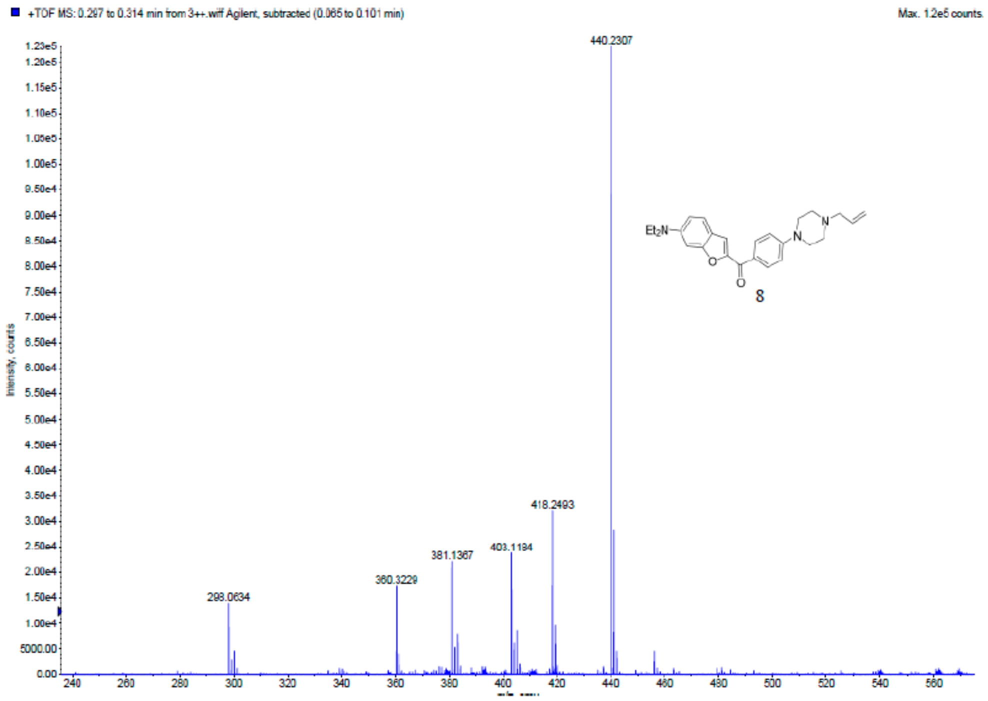

Elemental composition calculator

```
Target m/z:         +440.2307      amu
Tolerance:          +100.0000      ppm
Result type:       Elemental
Max num of results:       100
Min DBE:     -0.5000      Max DBE:          +50.0000
Electron state:   Even
Num of charges:   1
Add water:        N/A
Add proton:       N/A
File Name:        3++.wiff
```

	Elements	Min Number	Max Number
1	Br	0	0
2	C	0	26
3	F	0	0
4	H	0	31
5	Ir	0	0
6	N	0	3
7	Na	0	1
8	O	0	2
9	S	0	0

	Formula	Calculated m/z (amu)	mDa Error	PPM Error	DBE
1	C26 H31 N3 O2 Na	440.2308	-0.1486	-0.3376	12.5

Figure S47. HRMS spectrum of compound 8.

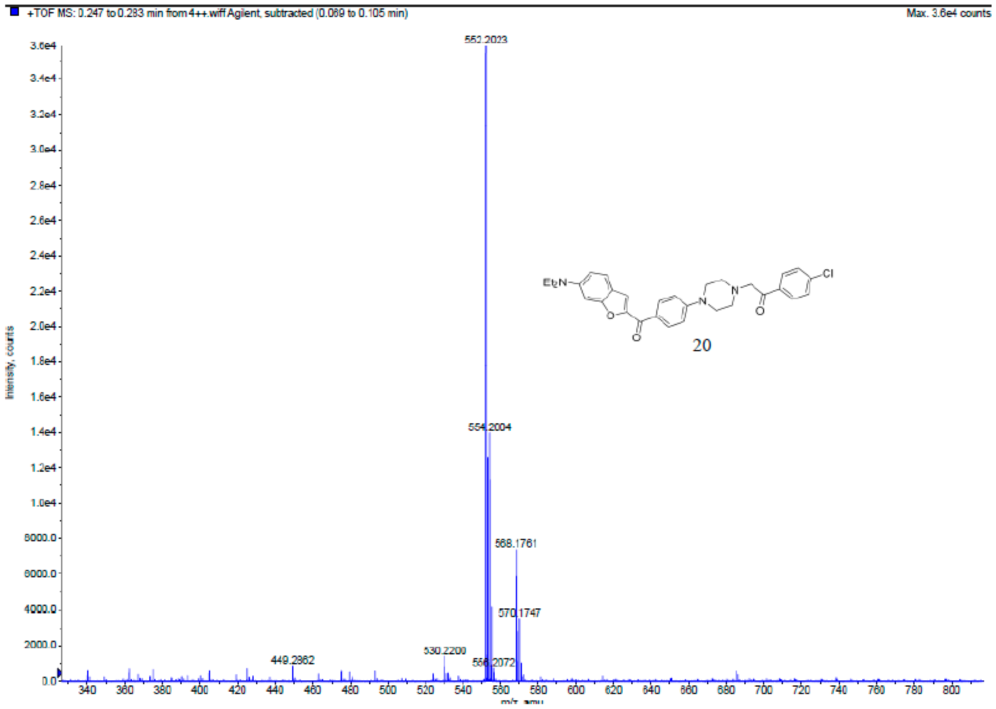

Elemental composition calculator

Target m/z: +552.2023 amu
Tolerance: +100.0000 ppm
Result type: Elemental
Max num of results: 100
Min DBE: −0.5000 Max DBE: +50.0000
Electron state: Even
Num of charges: 1
Add water: N/A
Add proton: N/A
File Name: 4++.wiff

	Elements	Min Number	Max Number
1	Cl	0	1
2	C	0	31
3	F	0	0
4	H	0	32
5	Ir	0	0
6	N	0	3
7	Na	0	1
8	O	0	3
9	S	0	0

	Formula	Calculated m/z (amu)	mDa Error	PPM Error	DBE
1	C31 H32 N3 O3 Na Cl	552.2024	−0.1410	−0.2554	16.5

Figure S48. HRMS spectrum of compound 20.

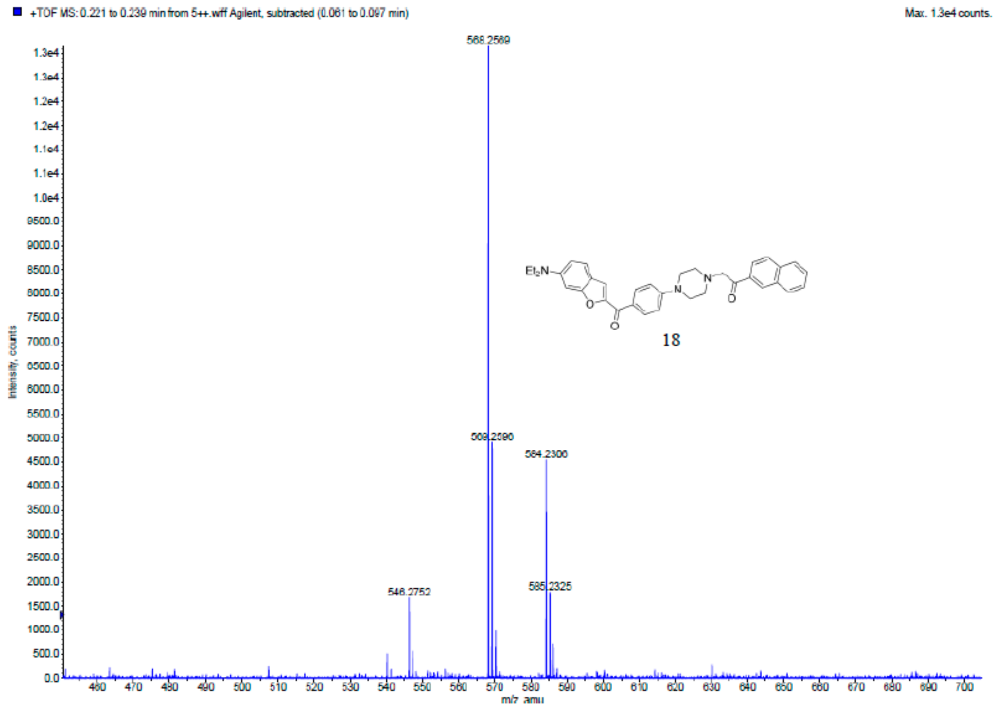

Elemental composition calculator

```
Target m/z:        +568.2569      amu
Tolerance:          +10.0000      ppm
Result type:      Elemental
Max num of results:       100
Min DBE:    -0.5000       Max DBE:          +50.0000
Electron state:   Even
Num of charges:   1
Add water:        N/A
Add proton:       N/A
File Name:        5++.wiff
```

	Elements	Min Number	Max Number
1	Cl	0	1
2	C	0	35
3	F	0	0
4	H	0	35
5	Ir	0	0
6	N	0	3
7	Na	0	1
8	O	0	3
9	S	0	0

	Formula	Calculated m/z (amu)	mDa Error	PPM Error	DBE
1	C35 H35 N3 O3 Na	568.2570	-0.1634	-0.2876	19.5

Figure S49. HRMS spectrum of compound 18.

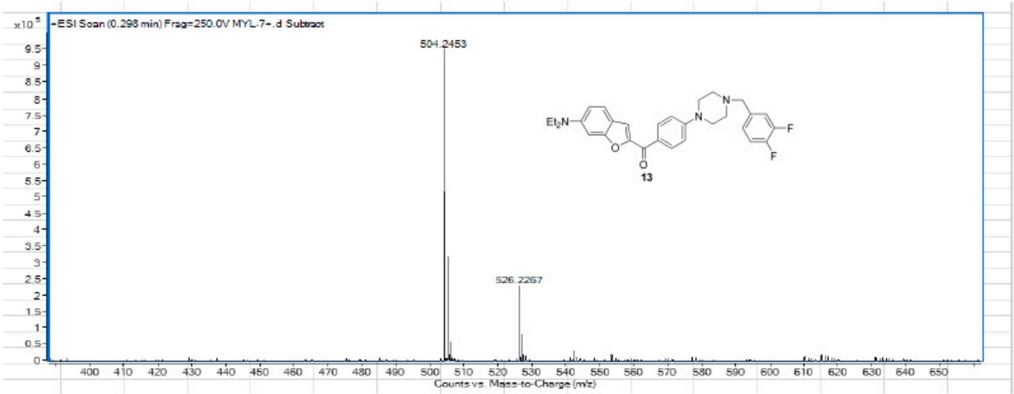

MS Formula Results: + Scan (0.298 min) Sub (MYL-7+.d)

m/z	Ion	Formula	Abundance
504.2453	(M+H)+	C30 H32 F2 N3 O2	652181.8

Best	Formula (M)	Ion Formula	Score	Cross Score	Calc m/z	Diff (ppm)	Mass Match	Abund Match	Spacing Match
✔	C30 H31 F2 N3...	C30 H32 F2 N3 O2	99.19		504.2457	0.92	99.07	99.91	98.55

Figure S50. HRMS spectrum of compound 13.

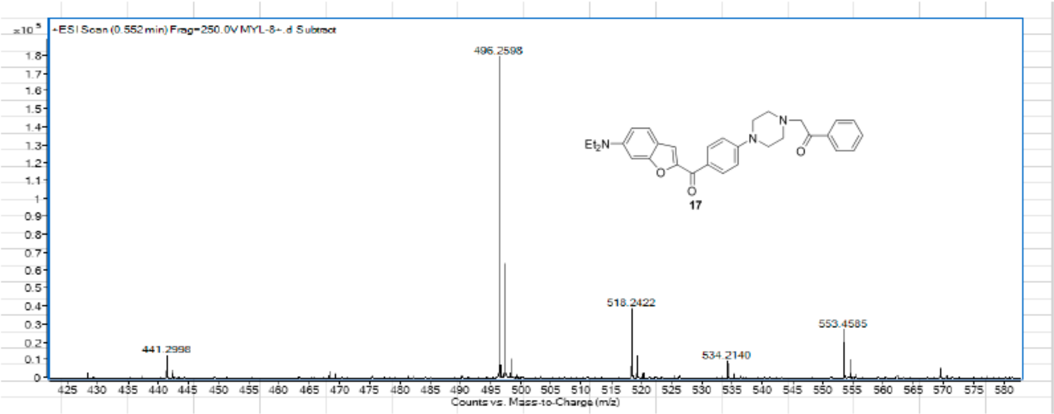

MS Formula Results: + Scan (0.552 min) Sub (MYL-8+.d)

m/z	Ion	Formula	Abundance
496.2598	(M+H)+	C31 H34 N3 O3	182070.2

Best	Formula (M)	Ion Formula	Score	Cross Score	Calc m/z	Diff (ppm)	Mass Match	Abund Match	Spacing Match
✔	C31 H33 N3 O3	C31 H34 N3 O3	98.98		496.2595	-0.78	99.36	99.49	97.62

Figure S51. HRMS spectrum of compound 17.

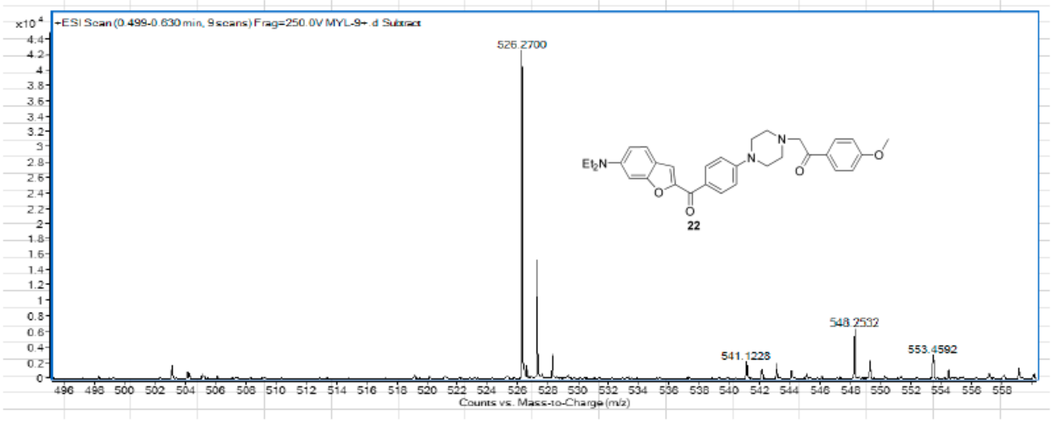

MS Formula Results: + Scan (0.499-0.630 min) Sub (MYL-9+.d)

m/z	Ion	Formula	Abundance
526.27	(M+H)+	C32 H36 N3 O4	42856.5

Best	Formula (M)	Ion Formula	Score	Cross Score	Calc m/z	Diff (ppm)	Mass Match	Abund Match	Spacing Match
✓	C32 H35 N3 O4	C32 H36 N3 O4	98.74		526.27	-0.04	100	99.82	94.92

Figure S52. HRMS spectrum of compound 22.

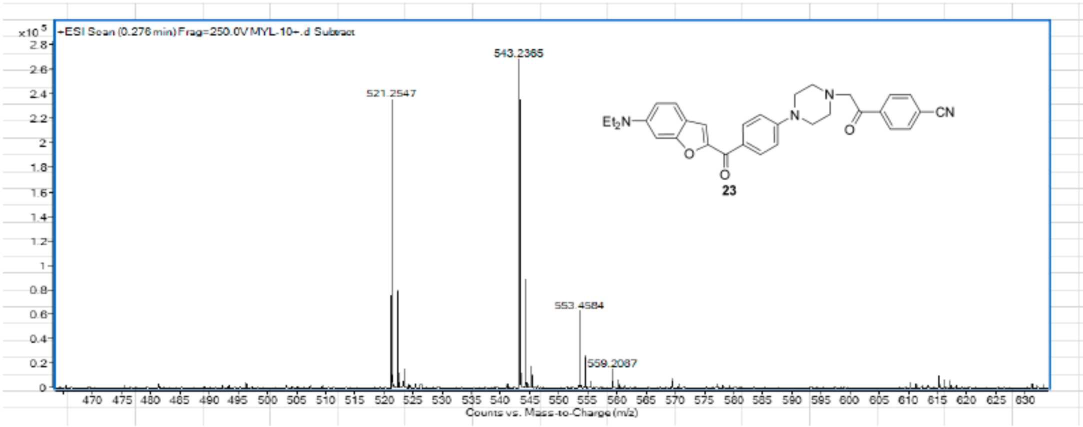

MS Formula Results: + Scan (0.276 min) Sub (MYL-10+.d)

m/z	Ion	Formula	Abundance
521.2547	(M+H)+	C32 H33 N4 O3	235941.4

Best	Formula (M)	Ion Formula	Score	Cross Score	Calc m/z	Diff (ppm)	Mass Match	Abund Match	Spacing Match
✓	C32 H32 N4 O3	C32 H33 N4 O3	99.08		521.2547	0.15	99.97	98.08	98.48

Figure S53. HRMS spectrum of compound 23.

Qualitative Analysis Report

**Data Filename**	xp-1.d	**Sample Name**	xp-1
**Sample Type**	Sample	**Position**	P1-B7
**Instrument Name**	Instrument 1	**User Name**	
**Acq Method**	SIBU.m	**Acquired Time**	5/3/2016 3:15:08 PM
**IRM Calibration Status**	Success	**DA Method**	ESI+.m
**Comment**			

Sample Group **Info.**

Acquisition SW 6200 series TOF/6500 series

Version Q-TOF B.05.01 (B5125.2)

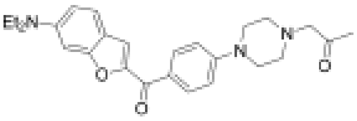

User Spectra

Fragmentor Voltage	Collision Energy	Ionization Mode
135	0	ESI

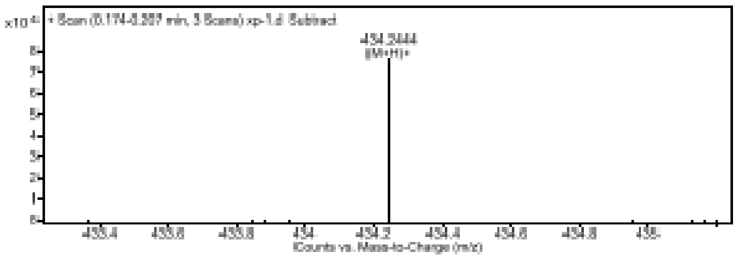

Peak List

m/z	z	Abund	Formula	Ion
217.6264	2	8952.2		
218.1264	2	2590.61		
434.2444	1	76264.91	C26 H31 N3 O3	(M+H)+
435.2475	1	21341.15	C26 H31 N3 O3	(M+H)+
436.2495	1	3163.94	C26 H31 N3 O3	(M+H)+
456.2261	1	11120.98		
457.2292	1	2948.91		
472.1999	1	7430.06		

Formula Calculator Element Limits

Element	Min	Max
C	3	60
H	0	120
O	0	30
N	0	10

Formula Calculator Results

Formula	CalculatedMass	CalculatedMz	Mz	Diff. (mDa)	Diff. (ppm)	DBE
C26 H31 N3 O3	433.2365	434.2438	434.2444	-0.6	-1.3	13.0000

--- End Of Report ---

Figure S54. HRMS spectrum of compound 16.

Qualitative Analysis Report

**Data Filename**	xp-2.d	**Sample Name**	xp-2
**Sample Type**	Sample	**Position**	P1-B8
**Instrument Name**	Instrument 1	**User Name**	
**Acq Method**	SIBU.m	**Acquired Time**	5/3/2016 3:16:30 PM
**IRM Calibration Status**	Success	**DA Method**	ESI+.m
**Comment**			

**Sample Group**		**Info.**
**Acquisition SW**	6200 series TOF/6500 series	
**Version**	Q-TOF B.05.01 (B5125.2)	

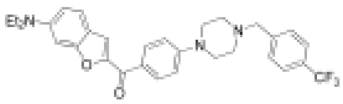

User Spectra

Fragmentor Voltage	Collision Energy	Ionization Mode
135	0	ESI

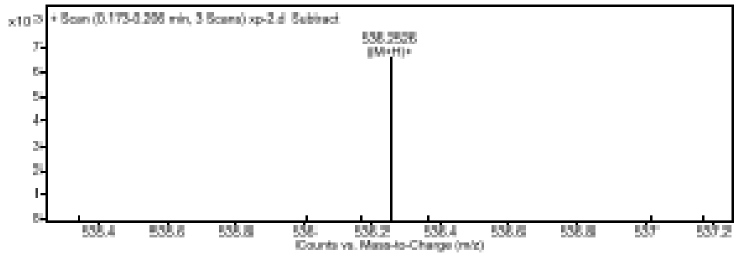

Peak List

m/z	z	Abund	Formula	Ion
121.0509	1	8854.64		
277.1775		1434.44		
536.2526	1	6988.54	C31 H32 F3 N3 O2	(M+H)+
537.254	1	2069.8	C31 H32 F3 N3 O2	(M+H)+
558.235	1	1422.66		
574.209	1	1774.92		
607.4705	1	1835.52		
922.0098		4875.69		

Formula Calculator Element Limits

Element	Min	Max
C	3	60
H	0	120
O	0	30
N	0	10
F	0	10

Formula Calculator Results

Formula	CalculatedMass	CalculatedMz	Mz	Diff. (mDa)	Diff. (ppm)	DBE
C31 H32 F3 N3 O2	535.2447	536.2519	536.2526	-0.3	-0.5	16.0000

--- End Of Report ---

Agilent Technologies Page 1 of 1 Printed at: 4:10 PM on: 5/3/2016

Figure S55. HRMS spectrum of compound 12.

Qualitative Analysis Report

**Data Filename**	xp-3.d	**Sample Name**	xp-3
**Sample Type**	Sample	**Position**	P1-B9
**Instrument Name**	Instrument 1	**User Name**	
**Acq Method**	SIBJ.m	**Acquired Time**	5/3/2016 3:17:50 PM
**IRM Calibration Status**	Success	**DA Method**	ESI+.m
**Comment**			

Sample Group **Info.**

Acquisition SW 6200 series TOF/6500 series

Version Q-TOF B.05.01 (B5125.2)

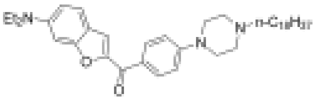

User Spectra

Fragmentor Voltage	Collision Energy	Ionization Mode
135	0	ESI

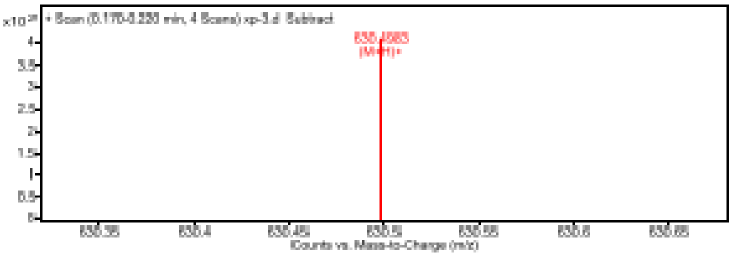

Peak List

m/z	z	Abund
274.2747	1	2374.47
318.3003	1	1604.12
358.3685	1	2149.94
359.2565	1	1377.72
391.1634	1	1478.04
424.2822	1	1844.82
536.2526	1	2189.1
607.4688		1698.78

Formula Calculator Element Limits

Element	Min	Max
C	3	60
H	0	120
O	0	30
N	0	10

Formula Calculator Results

Formula	CalculatedMass	CalculatedMz	Mz	Diff. (mDa)	Diff. (ppm)	DBE
C41 H63 N3 O2	629.4920	630.4993	630.4983	-0.7	-1.1	12.0000

--- End Of Report ---

Agilent Technologies Page 1 of 1 Printed at: 9:16 AM on: 5/4/2016

Figure S56. HRMS spectrum of compound 7.

Qualitative Analysis Report

**Data Filename**	xp-4.d	**Sample Name**	xp-4
**Sample Type**	Sample	**Position**	P1-C1
**Instrument Name**	Instrument 1	**User Name**	
**Acq Method**	SIBU.m	**Acquired Time**	5/3/2016 3:19:11 PM
**IRM Calibration Status**	Success	**DA Method**	ESI+.m
**Comment**			

Sample Group **Info.**

Acquisition SW 6200 series TOF/6500 series

Version Q-TOF B.05.01 (B5125.2)

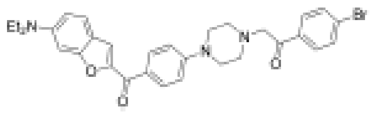

User Spectra

Fragmentor Voltage	Collision Energy	Ionization Mode
135	0	ESI

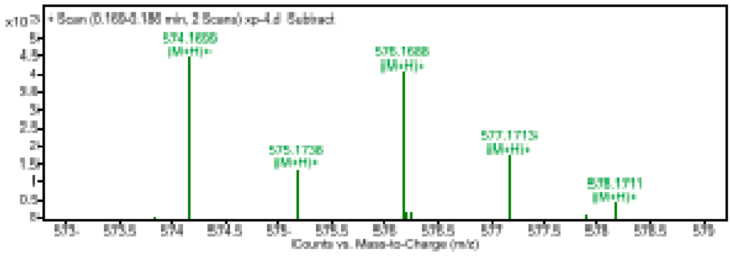

Peak List

m/z	z	Abund	Formula	Ion
294.2066		1246.78		
574.1699	1	4464.83	C31 H32 Br N3 O3	(M+H)+
575.1738	1	1313.03	C31 H32 Br N3 O3	(M+H)+
576.1688	1	4028.82	C31 H32 Br N3 O3	(M+H)+
577.1713	1	1702.03	C31 H32 Br N3 O3	(M+H)+
607.4672	1	1181.44		
614.1236		1438.36		
922.0098		1498.96		

Formula Calculator Element Limits

Element	Min	Max
C	3	60
H	0	120
O	0	30
N	0	10
Br	0	5

Formula Calculator Results

Formula	CalculatedMass	CalculatedMz	Mz	Diff. (mDa)	Diff. (ppm)	DBE
C31 H32 Br N3 O3	573.1627	574.1700	574.1699	-0.1	-0.1	17.0000

--- End Of Report ---

Agilent Technologies Page 1 of 1 Printed at: 9:14 AM on: 5/4/2016

Figure S57. HRMS spectrum of compound 21.

Qualitative Analysis Report

**Data Filename**	xp-5.d	**Sample Name**	xp-5
**Sample Type**	Sample	**Position**	P1-C2
**Instrument Name**	Instrument 1	**User Name**	
**Acq Method**	SIBU.m	**Acquired Time**	5/3/2016 3:20:33 PM
**IRM Calibration Status**	Success	**DA Method**	ESI+.m
**Comment**			

Sample Group **Info.**

Acquisition SW 6200 series TOF/6500 series

Version Q-TOF B.05.01 (B5125.2)

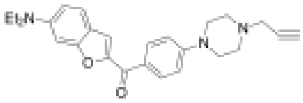

User Spectra

Fragmentor Voltage	Collision Energy	Ionization Mode
135	0	ESI

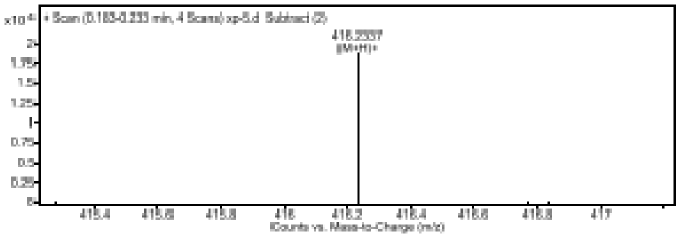

Peak List

m/z	z	Abund	Formula	Ion
359.257	1	1765.73		
407.1378		1684.78		
416.2337	1	187712.71	C26 H29 N3 O2	(M+H)+
417.2373	1	5685.55	C26 H29 N3 O2	(M+H)+
438.2156	1	3498.21		
454.1889	1	3801.03		
454.2494	1	6063.16		
455.2515	1	2248.17		

Formula Calculator Element Limits

Element	Min	Max
C	3	60
H	0	120
O	0	30
N	0	10

Formula Calculator Results

Formula	CalculatedMass	CalculatedMz	Mz	Diff. (mDa)	Diff. (ppm)	DBE
C26 H29 N3 O2	415.2260	416.2333	416.2337	-0.5	-1.2	14.0000

--- End Of Report ---

Agilent Technologies Page 1 of 1 Printed at: 9:15 AM on: 5/4/2016

Figure S58. HRMS spectrum of compound 9.

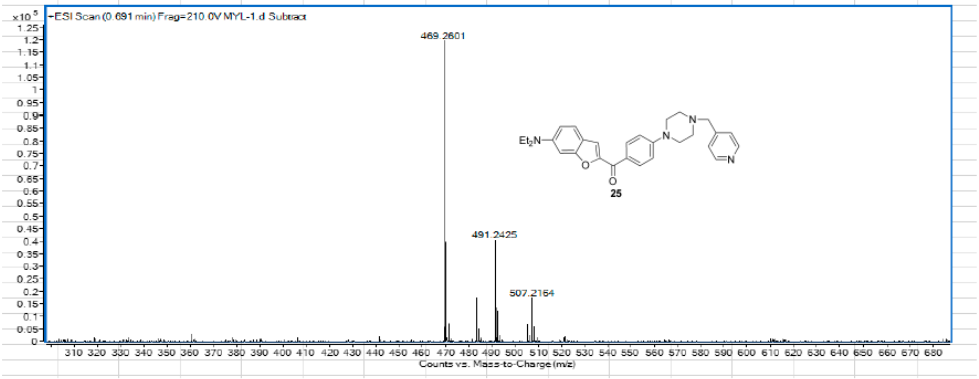

MS Formula Results: + Scan (0.691 min) Sub (MYL-1.d)

m/z	Ion	Formula	Abundance
469.2601	(M+H)+	C29 H33 N4 O2	121334.3

Best	Formula (M)	Ion Formula	Score	Cross Score	Calc m/z	Diff (ppm)	Mass Match	Abund Match	Spacing Match
✔	C29 H32 N4 O2	C29 H33 N4 O2	99.14		469.2598	-0.7	99.5	99.37	98.14

Figure S59. HRMS spectrum of compound 25.
